# Refeeding with different levels of dietary carbohydrate modulates epigenetic stability through global DNA (de)methylation and histone modifications in juvenile and adult Nile tilapia (*Oreochromis niloticus*)

**DOI:** 10.1080/15592294.2025.2566514

**Published:** 2025-09-29

**Authors:** Sirijanya Thongchaitriwat, Suksan Kumkhong, Cécile Heraud, Karine Dias, Stephane Panserat, Surintorn Boonanuntanasarn, Lucie Marandel

**Affiliations:** a School of Animal Technology and Innovation, Institute of Agricultural Technology, Suranaree University of Technology, Nakhon Ratchasima, Thailand; b INRAE, Université de Pau et des Pays de l’Adour, NuMéA, Saint-Pée-sur-Nivelle, France

**Keywords:** Refeeding, carbohydrate, 5-methylcytosine, histone, *oreochromis niloticus*

## Abstract

The Nile tilapia (*Oreochromis niloticus*) exhibits a strong metabolic response to dietary carbohydrates (CHO). Short-term refeeding after fasting with a high-carbohydrate (HC) diet has been shown to modulate CHO metabolism, but the role of epigenetic regulation in this response remains unclear. This study investigated how short-term refeeding with either a HC [639.2 g kg^−1^ diet]/low-protein [164.9 g kg^−1^ diet] (HC/LP) diet or a low-CHO [47.4 g kg^−1^ diet]/high-protein [607.9 g kg^−1^ diet] (LC/HP) diet influences global DNA methylation and demethylation, histone modifications, and mRNA levels of epigenetic regulators in the liver and muscle of juvenile and adult Nile tilapia. Following a 4-day fasting period, fish were refed for 4 days with either HC/LP or LC/HP diets. Compared to the fasted state, refeeding with either diet altered epigenetic markers by: (1) decreasing hepatic global DNA 5-mC oxidative derivatives—5-hmdC in juveniles, and both 5-hmdC and 5-cadC in adults; (2) inducing histone hypermethylation and/or hyperacetylation – H3K9ac (hepatic) and H3K36me3 (muscular) in juveniles, and H3K9me3 and H3K9ac (muscular) in adults; and (3) promoting expression of enzymes related to DNA hypermethylation (upregulated *dnmt*, downregulated *tet*) and histone hypermethylation/acetylation (upregulated *setd1b*, *kmt2, suv39h1b*; downregulated *kdm4, sirt5*). Diet-specific effects included hepatic H3K36 hypomethylation and H3K9 hypoacetylation in juveniles fed HC/LP, accompanied by upregulation of *kdm4b, kdm4c*, and *sirt5*. In adults, HC/LP refeeding induced muscular DNA hypomethylation and H3K9 hypoacetylation, associated with upregulation of *tet, sirt2*, and *sirt5*. Refeeding following fasting induced histone hypermethylation and/or hyperacetylation, while HC refeeding was particularly associated with muscular global DNA hypomethylation and histone hypoacetylation/methylation.

## HIGHLIGHTS

- Understanding epigenetic responses to diet can inform sustainable aquaculture practices by optimizing feed composition for health, performance, and adaptability.
- Short-term refeeding with diets varying in carbohydrate and protein levels modulates key epigenetic mechanisms – including DNA methylation and histone modifications – in Nile tilapia.
- High-carbohydrate/low-protein (HC/LP) refeeding induces tissue-specific hypomethylation and hypoacetylation, particularly in muscle, which may have long-term implications for metabolic health and growth.
- Epigenetic responses to refeeding differ between life stages, emphasizing the need for age-specific feeding strategies in aquaculture.
- Nutritional programming through short-term dietary interventions may influence gene regulation beyond immediate metabolic effects, supporting precision feeding approaches.

## Introduction

Epigenetics refers to the study of how environmental or behavioural factors can modify gene activity without altering the underlying DNA sequence [1]. Among these factors, nutritional status plays a key role in regulating metabolism, and this regulation may be mediated by epigenetic mechanisms – particularly DNA methylation/demethylation and histone modifications – in animals [2–6]. For example, in mammals [7,8], DNA methylation was influenced by the metabolic cofactors during nutritional status, which could impact the changes in the dynamic balances of DNA methylation modulators (both writers, DNA methyltransferase family DNMTs, and erasers, the Ten-eleven translocation (TET) enzyme family). In mice, time-restricted feeding has been shown to alter DNMT and TET expression at both protein and transcript levels in the brain and liver [9,10]. Furthermore, nutritional status can establish a form of metabolic memory by reshaping the epigenetic landscape, in part by altering chromatin structure [2]. Histone modifications, such as methylation and acetylation, are key epigenetic mechanisms involved in chromatin remodelling and regulation of gene expression [11]. For example, in human cell cultures, glucose restriction induced changes in histone marks at gene promoter regions, leading to transcriptional alterations in target genes [12]. Moreover, in teleost fish, although histone modifications at gluconeogenic gene loci were not affected by nutritional status or dietary carbohydrate (CHO) in juvenile rainbow trout, global hepatic hypermethylation of H3K9 during refeeding and global hepatic hyperacetylation under no-CHO conditions were observed [2]. Additionally, activation of *pepck* mRNA expression was associated with upregulation of hepatic H3K4me3 at the *pepck* promoter region, which may have contributed to hyperglycaemia and anorexia in mandarin fish fed CHO-rich diets [13]. These histone modifications, along with active DNA methylation mechanisms, are regulated by a variety of enzymes: histone methylation writers (histone lysine methyltransferases, KMTs), erasers (histone lysine demethylases, KDMs), acetylation writers (histone lysine acetyltransferases, KATs), and erasers (histone deacetylases or sirtuins, SIRTs).

Previous studies have demonstrated that specific dietary nutrients (e.g., refeeding, CHO, and/or protein) can influence the epigenetic landscape, affecting DNA methylation, histone modifications, and epigenetic modulators in both mammals [14], and fish [2,3]. In rainbow trout, refeeding after a period of fasting has been shown to alter the hepatic epigenetic landscape, including changes in histone modifications, DNA hypomethylation, and the transcript levels of associated epigenetic modulators [2,3]. This hypomethylation is thought to occur via the active DNA demethylation pathway, mediated by TET enzymes, which iteratively oxidize 5-methylcytosine (5-mC) to generate the oxidative derivatives 5-hydroxymethylcytosine (5-hmC), 5-formylcytosine (5-fC), and 5-carboxylcytosine (5-caC), followed by thymine DNA glycosylase (TDG)-dependent base excision repair or replication-dependent dilution [15–17], ultimately restoring cytosine. Specifically, the high-CHO (HC) and low-protein (LP) diets increased hepatic levels of 5-hydroxymethyl-2’-deoxycytidine (5-hmdC), while either the LP or HC diet independently decreased levels of 5-methyl-2′-deoxycytidine (5-mdC), compared with the control diet [3]. It is increasingly important to consider these oxidative intermediates, as accumulating evidence suggests that they are dynamic, may possess regulatory functions [18–21] and might even represent stable DNA modifications [22].

Rainbow trout, a carnivorous fish, is often considered a model of ‘poor utilization’ of dietary CHO. Hepatic epigenetic remodelling induced by dietary CHO – both at the global level and at gluconeogenesis-related gene loci – has been suggested to play a key role in the nutritionally glucose-intolerant phenotype observed in this species [2]. In contrast, Nile tilapia (*Oreochromis niloticus*), an omnivorous freshwater species, is regarded as a ‘highly adaptable user’ of dietary CHO, efficiently utilizing it as a primary energy source [23]. While this metabolic flexibility has been well studied at physiological, biochemical, and transcriptional levels across different life stages [23–27] the underlying mechanisms – particularly at the epigenetic level – remain largely unexplored. Understanding how fasting and refeeding with varying dietary CHO/protein (CHO/CP) ratios affect the epigenetic landscape in a species known for effective CHO utilization is critical. First, it could reveal mechanisms that underpin Nile tilapia’s efficient dietary CHO use. Second, such insights may support improvements in CHO utilization in other aquaculture-relevant species, such as salmonids. Therefore, the present study aims to investigate how fasting and subsequent refeeding with diets differing in CHO/CP ratios influence the epigenetic landscape in Nile tilapia, focusing on DNA methylation (including oxidative derivatives) and histone modifications known to be CHO-responsive [2], as well as the expression of associated epigenetic modulators (e.g., mRNA levels of writer and eraser enzymes). Since environmental adaptability may be linked to physiological stage – as previously shown in trout [28]—this study focuses on two key life stages: juvenile and adult.

## Material and methods

### Experimental design, diet, and fish culture

**Figure 1.**
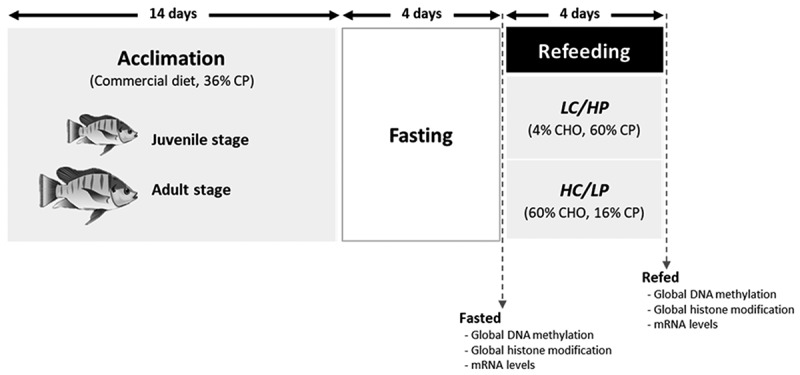
Experimental plan for fasting and refeeding juvenile and adult Nile tilapia. Fish were fed a commercial diet during the acclimation period. After fasting for 4 days, fish were subsequently refed for 4 days with either a low-carbohydrate/high-protein (LC/HP) or high-carbohydrate/low-protein (HC/LP) diets.

The samples in this manuscript were obtained from a previously published article by Thongchaitriwat et al. [29]. The experimental plan for fasting and subsequent refeeding, which was randomised with six replicates (tanks), is shown in Figure 1. Nile tilapia efficiently utilizes CHO as an economical energy source to support protein-sparing growth [30]. Therefore, HC/LP and LC/HP diets are commonly used to investigate the effects of dietary CHO levels while maintaining a fixed gross energy content in the diet. In this study, refeeding diets including HC/LP and LC/HP diet were formulated (Table 1). The experimental diets and their proximate compositions, including moisture, CP, CF, crude fibre, and ash, which were analysed according to the standard method of the Association of Official Analytical Chemists [31], are listed in Table 1.

**Table 1.** Ingredients and chemical composition (g kg^−1^) of the commercial diet (used during acclimation) and the refeeding diets: low-carbohydrate/high-protein (LC/HP) and high-carbohydrate/low-protein (HC/LP).

Ingredients	Acclimation diet		Refeeding diets	
	Commercial diet		LC/HP	HC/LP
Fish meal	–		880	180
Rice flour	–		0	700
Fish oil	–		0	70
Soybean oil	–		20	0
Gelatin	–		80	0
Di-calcium phosphate	–		0	30
Fish premix^a^	–		20	20
	**Proximate composition (g kg**^**−1**^ **dry weight)**			
Dry matter	916.4		942.1	921.9
Protein	393.0		607.9	164.9
Fat	36.8		97.9	98.5
Fiber	77.7		5.2	4.6
Ash	137.0		241.6	92.9
NFE^b^	355.4		47.4	639.2
Gross energy (kJ g^−1^)	13.13		14.40	15.6

Abbreviations: LC/HP = low carbohydrate and high protein; HC/LP = high carbohydrate and low protein.

^a^Vitamin and trace mineral mix provided the following (IU kg − 1 or g kg − 1 diet): biotin, 0.25 g; folic acid, 0.003 g; inositol, 0.25 mg; niacin, 0.0215 g; pantothenic acid, 0.03 g; vitamin A, 5,000 IU; vitamin B1, 0.0025 g; vitamin B2, 0.0012 g; vitamin B6, 0.0075 g; vitamin B12, 0.00005 mg; vitamin C, 1 g; vitamin D3, 1,000 IU; vitamin E, 100 IU; vitamin K, 0.008 g; copper, 0.02 g; iron, 0.2 g; selenium, 0.3 mg; zinc, 0.32 g.

^b^Nitrogen-free extract (g kg^−1^ dry weight) = 1,000 − (CP + crude lipid + crude fibre + ash).

The experiment was conducted with six replicates of the pond per condition (i.e., LC/HP and HC/LP refeeding fish). During the experimental trial, 60 juvenile fish (50–60 g) were randomly distributed in cement tanks (4 m^2^, water depth: 0.8 m) under continuous aeration. For the second experiment, 34 adult fish (450–550 g) were randomly distributed in cement tanks (water depth: 0.8 m) under continuous aeration. A flow-through water change system was implemented by replacing one-third of the water in each tank weekly with dechlorinated water.

Before the experimental trial, fish were acclimatized to the experimental conditions for 14 days, and fish were hand fed twice per day (9.00 and 16.00) with a commercial diet (36% crude protein [CP] + 4% crude fat [CF]) administered at 3% of their body weight. Subsequently, fish were randomly divided into two groups according to the experimental refeeding diets, including LC/HP and HC/LP diets (*n* = 6 replicates; juvenile; 22 fish/replication, adult: 13 fish/replication). After acclimation, fish were fed-deprived for 4 days (fasted). During refeeding, fish were refed at 3% of their body weight with either the HC/LP or LC/HP diet for four days.

Throughout the experimental period, air and water temperatures were determined daily and ranged from 29.0°C to 31.0°C and 27.0°C to 28.0°C, respectively. Dissolved oxygen (DO) and pH were measured daily using a DO and pH meter, and their values were within acceptable ranges of 4.30–5.58 mg L^−1^ and 7.49–8.71, respectively. During the experimental period, fish deaths were recorded to determine their survival rates, and no mortality was observed.

### Fish sampling

Samples were collected after 4 days of fasting (fasted) and 4 days of refeeding periods (refed) (Figure 1). For refed conditions, fish were sampled based on the postprandial glycaemia curve of Nile tilapia determined in a previous study [23]. At each sampling point, fish (*n* = 6 replicates; juvenile: 4 fish/replication, adult: 2 fish/replication) were euthanised using 0.2% clove oil. After fish bleeding, liver and epaxial muscle samples were collected, snap-frozen in liquid nitrogen, and stored at −80°C for analysis of global DNA demethylation, global histone modification, and mRNA levels of genes involved in epigenetic modifications.

### Total RNA extraction, cDNA synthesis, and real-time RT-PCR analysis of genes involved in epigenetic modifications

Expression of genes related to epigenetic modification was examined in the liver and muscle samples after four days of fasting (fasted), and four days of refeeding (refed) with either LC/HP or HC/LP diet (at each sampling point: two fish/replicate; *n* = 6 replicates). Total RNA was extracted from the liver (50 mg) and muscle (100 mg) samples using the TRIzol reagent (Catalogue number: 15596026, Invitrogen, Carlsbad, CA, USA). The quantity of total RNA was measured using a NanoDrop spectrophotometer (Thermo Fisher, Madison, WI, USA), and the integrity of the total RNA was verified by 1% agarose gel electrophoresis.

DNA sequences of enzymes related to DNA (de)methylation, histone (de)methylation, and (de)acetylation were retrieved using https://www.ensembl.org/index.html and https://www.ncbi.nlm.nih.gov/nucleotide (Table 2). To validate the nucleotide sequences, primers were designed and reverse transcription polymerase chain reaction (RT-PCR) was performed. The size of PCR products was verified by 2% agarose gel electrophoresis, and all PCR products were analysed for their nucleotide sequences (Supplementary Table S1; https://doi.org/10.7910/DVN/S0PAOF). The primer sequences of gene-related epigenetic modulators and the reference gene [32] used for real-time RT-qPCR are listed in Table 2.

**Table 2.** List of primers used for RT-qPCR.

Genes		5‘/3’ Forward primer	5‘/3’ Reverse primer	SIZE (bps)	Accession number	
					ENSEMBL	NCBI
Reference gene	ef1*	GCACGCTCTGCTGGCCTTT	GCGCTCAATCTTCCATCCC	250	ENSONIG00000035055	AB075952
DNA methylation writers	*dnmt1*	CTCACACTGCGCTGTCTTGT	ACAACGCTGAGAGAGCAAGC	188	ENSONIG00000001574	XM_025906327.1
	*dnmt3aa*	CCAACAACCACGAGCAGGAA	TGCCGACAGTGATGGAGTCT	192	ENSONIG00000005542	XM_005475084.4
	*dnmt3ab*	GCCGCAGCTTAGAGGACATC	CACACATGAGCACCTCTCGTC	189	ENSONIG00000001050	XM_005477258.3
	*dnmt3ba*	GCTGCTGCAGATGCTACTGT	TTGCGCTGTTGTTGGCAAAG	186	ENSONIG00000016781	XM_025901732.1
	*dnmt3bb*	TGCAGGAGTTCTTCGCCAAC	TGCCACATACTGACCCACCT	173	ENSONIG00000014841	XM_025901790.1
DNA methylation erasers	*tet1*	CATCCAGTCCCAGCACAACC	CTCTATTTGGCGTGCGCTGA	194	ENSONIG00000033407	XM_025897345.1
	*tet2*	GCAGCTGCCAACAAGAATGC	TGTTGCTGCTGCTGATGGAC	191	ENSONIG00000028706	XM_005457001.3
	*tet3*	GCAAGCCAACCAACCAAACC	GATGTGTTGGCTCCGACCTG	177	ENSONIG00000015843	XM_019365521.2
H3K4me3 writers (Histone Lysine methyltransferase)	*setd1a*	GGAACTCCGGTCTGGATGGT	CGAAGCTGCCCATCTGTGTT	172	ENSONIG00000009854	XM_005468973.4
	*setd1ba*	AAGACAGGGAGGCAGCAGAA	CCTCAGGACTGGGAGGTCTG	198	ENSONIG00000013898	XM_005470275.4
	*kmt2a*	AGAGCAGGAAAGCCAACAGC	CACTGGGCGTAGTTGTGGTC	178	ENSONIG00000006229	XM_013274782.3
	*kmt2ba*	ACTCTGAGGGACCTGGAGGA	AGAGGAGGTGAAGCCGATCC	191	ENSONIG00000019760	XM_013275905.3
	*kmt2bb*	GCTCCCGTCAGTGTGTCTTC	TCTGGCTCCAACCCAGTCAA	172	ENSONIG00000002752	XM_013277028.3
H3K4me3 erasers (Histone lysine demethylases)	*kdm5a*	TCTGGCCACAGAGGAGTTGT	GTGACGTGGCTCTGCTGAAA	191	ENSONIG00000009761	XM_005451728.4
	*kdm5ba*	TCTCAGAGCAGAGGGCATCC	GACCCGATGTCACACCTTGG	165	ENSONIG00000019223	XM_003441348.2
	*kdm5bb*	CATCCCTGCCTACCTCCCAA	AAGGCTCCAGGTGGACTTGA	170	ENSONIG00000016770	XM_003439103.5
	*kdm5c*	CTCTCCACCCTGGAGGCAAT	AGCTACCAGGCCCTCCAAAT	174	ENSONIG00000016838	XM_005448517.4
	*riox1*	CCACCTGGCACACAAGGATT	TCCGGCTTCTACCACCACAT	192	ENSONIG00000005898	XM_005475002.4
H3K9me3 specific writer	*suv39h1b*	TCCAACGCATGGCCTACAAC	CTTGATGTGCTGCAGTGTGC	197	ENSONIG00000011649	XM_003459875.5
H3K9me3 and H3K36me3 erasers	*kdm4aa*	CGGATGCGAACCAAACCTCT	GGCTGGATCGACACCGTAAC	180	ENSONIG00000007297	XM_005457300.3
	*kdm4ab*	TCTGTTCAGGGAGGCACACA	GCCTGTTGGCCCATCTGTTT	162	ENSONIG00000010525	XM_005476068.4
	*kdm4b*	TGCTCGCTCTTCTGTCCGTA	AGCAGATCAGGAGGCTGGTT	196	ENSONIG00000012580	XM_005453970.4
	*kdm4c*	CCTGCAGAGGAATGCAGTGG	GCACAGGTGCAATCTGGTGA	176	ENSONIG00000008824	XM_005456806.2
H3K36me3 specific writer	*setd2*	AGGCAGCGATGACTTCAAGC	ATCTTGTGGCGTCCCACTCT	182	ENSONIG00000003077	XM_019364854.2
H3K9ac specific writers	*kat2a*	CACTGACCCTGCTGCTATGC	GTAGGCCAACCAGCCACATC	173	ENSONIG00000001102	XM_025906390.1
	*kat2b*	GGCCTTTCATGGAGCCTGTG	CTCGCTCTCTGGAGGGTTGT	188	ENSONIG00000002420	XM_003444058.3
	*kat6a*	CATCCCGTCCACTGCTTTCC	CCTGTTCACGCTACCACCAC	173	ENSONIG00000015837	XM_005472980.3
	*gtf3c4*	CTTGTGGCGGTTCAAGCTCT	GGCTCGCCTTCCTCTTTCAC	174	ENSONIG00000013016	XM_003440231.5
H3K9ac specific erasers	*sirt2*	GCGAGTCTAGTCAGCAGGGT	CCCAGAAGATCAGCTAGAGCCA	197	ENSONIG00000016619	XM_003449264.5
	*sirt5*	ATTTGCCCAGGTGTGAGCAG	GAGCAAACATGGCTGCAGGA	177	ENSONIG00000006380	XM_003457306.5

*From Yang et al. [32].

To measure the mRNA levels of genes involved in epigenetic modification in liver and muscle tissue, quantitative real-time reverse-transcription polymerase chain reaction (real-time RT-qPCR) was performed. cDNA was synthesized from 1 µg of total RNA using SuperScript™ III Reverse Transcriptase (Invitrogen, USA; cat. no. 18080044), RNaseOUT™ (Invitrogen, USA; cat. no. 10777019), dNTP mix (Promega, France; cat. no. U1511), and random primers (Promega, France; cat. no. C1181), following the manufacturer’s protocol. Reverse transcription (duplicate for each sample) was performed, and each PCR assay (duplicate for each PCR reaction) was performed to analyse the mRNA level. Both hepatic and muscular tissues were examined for DNA methylation-related genes, including DNA methyltransferase type 1 (*dnmt1*) and DNA methyltransferase type 3 family members (*dnmt3aa*, *dnmt3ab*, *dnmt3ba*, and *dnmt3bb*). To evaluate the expressions of ten-eleven translocation (*tet1, tet2*, and *tet3*) involved in the deoxygenation of methyl-cytosine was determined. To investigate the regulation of histone modification of H3K4me3 writer, the genes that regulate histone lysine methyltransferase, including histone-lysine N-methyltransferase SETD1A (*setd1a*), histone-lysine N-methyltransferase SETD1B-A (*setd1ba*), lysine methyltransferase 2A (*kmt2a*), and histone-lysine N-methyltransferase 2B (*kmt2ba* and *kmt2bb*), were determined. Likewise, genes involved in the H3K4me3 eraser, including histone lysine demethylase 5 (*kdm5a, kdm5ba, kdm5bb*, and *kdm5c*) and bifunctional lysine-specific demethylase and histidyl hydroxylase (*riox1*), were also identified. In addition, H3K9me3 specific writer (histone lysine methyltransferase; *suv39h1b*) and H3K9me3 and H3K36me3 specific eraser (histone lysine demethylases 4; *kdm4aa, kdm4ab, kdm4b* and *kdm4c*) were investigated. The expression of SET domain-containing 2, a histone lysine methyltransferase (*setd2*) gene, involved in H3K36me3-specific writer, was determined. Moreover, the expression of genes involved in H3K9ac-specific writer (histone lysine acetyltransferase; *kat2a, kat2b, kat6a*, and general transcription factor IIIC subunit 4; *gtf3c4*) and H3K9ac-specific eraser (sirtuin; *sirt2* and *sirt5*) was detected. For the analysis of mRNA expression, the relative quantification of target gene expression was performed using the Roche Applied Science E-Method, as described by Pfaffl [33]. The relative mRNA level of elongation factor 1 alpha (*ef1α*) was used for normalising the measured mRNA in each tissue, as its relative expression did not change significantly over the sampling process (data not shown). In all cases, PCR efficiency was measured from the slope of a standard curve using serial dilutions of cDNA. In all cases, PCR efficiency values ranged between 1.8 and 2.0.

### Global DNA methylation and its demethylation derivatives by HPLC-UV

#### Non oxidant DNA extraction

Ten micrograms of tissue were added to 800 µL of 1 M Guanidine Thiocyanate (GTH) buffer and 10 µL of 0.25 mg/ml Proteinase K was added. Then, add the antioxidants, including 8 µL of 0.1 mM deferoxamine, 32 µL of 16 mM histidine and 8 µL of 3 mM glutathione. Samples were incubated in a dry bath at 57°C for 1 h and vortexed every 20 min. After incubation, samples were added 800 µL of Chloroform-alcohol isoamyl (24:1) and an antioxidant solution. Mixed samples were rotated on the rotating wheel for 15 min at room temperature. Then, samples were centrifuged at 10,000 rpm for 15 min at room temperature. Then, 300 µL of the aqueous phase from the samples was collected and transferred to a new tube. Then, samples were added with 75 µL of 5 M NaCl and 937 µL of absolute ethanol. Samples were mixed and incubated at −20°C for 15 min to precipitate DNA fragments. After incubation, samples were centrifuged at 4°C and 10,000 rpm for 15 min. After that, the ethanol was discarded, and the DNA pellet was washed with 1 mL of 70% ethanol. Then, the samples were centrifuged at 4°C and 10,000 rpm for 10 min. DNA samples were discarded with 70% ethanol by pipetting, and then the samples were dried in a dry bath at 45°C for 10 min or until the pellet was completely dry. DNA samples were cooled for 5 min before adding 150 µL of distilled water to dissolve the pellet, as described in the publication by Liu et al. [3]. Then, DNA samples were treated with RNase cocktail (Invitrogen) to remove RNA contamination. The quantity of DNA was measured using a NanoDrop spectrophotometer (Thermo Fisher, Madison, WI, USA).

#### DNA hydrolysis

One microgram of DNA was degraded into single nucleosides using the DNA Degradase Plus^TM^ kit (ZYMO RESEARCH) according to the manufacturer’s procedures. The DNA samples were prepared for mixture, including 1 µg of DNA (measured by Nanodrop), 2.5 µL of 10X DNA Degradase Plus^TM^ Reaction Buffer, 1 µL of DNA Degradase Plus^TM^ (5 units/µL), and ultrapure H2O to yield a total volume of 25 µL. The reaction will be mixed and incubated at 37°C for 2 h, followed by heat inactivation at 70°C for 20 min, as described in the publication by Liu et al. [3].

#### High-performance liquid chromatography-ultraviolet (HPLC-UV)

Nucleosides were separated, detected, and quantified using an HPLC-UV technique (Alliance, Waters Corporation). The separation was performed using a Luna C8, 5 µm, 150 × 4.6 mm, 100 Å LC column (Phenomenex). The compositions of the mobile phase will include solvent A, 10 mM potassium phosphate buffer, pH 3.7, and solvent B, 100% methanol. Linear gradient elution will be as follows: 0–8.5 min, 98% A; 8.5–11.8 min, 97% A; 11.8–18.9 min, 73% A; 18.9–21.2 min, 65% A. The temperature of the column oven was set at 30°C. The wavelength of UV detection will be 277 nm. The standards of 2 2’-deoxycytidine (dC), 5-methyl-2’-deoxycytidine (5-mdC), 5-hydroxymethyl-2’-deoxycytidine (5-hmdC), 5-formyl-2’-deoxycytidine (5-fdC) and 5-carboethoxy-2’-deoxycytidine (5-cadC) will be made using products from Berry & Associates Inc. Injection was done with either 20 µL of hydrolysed samples or standards. The identification of nucleosides will be based on retention times, as reported by Liu et al. [3].

#### Calculation of global 5-mdC and its other demethylation derivatives

The global level of dC, 5-mdC, 5-hmdC, 5-fdC and 5-cadC was calculated as a percentage of each molar quantity divided by the total of molar quantities of all detected cytidine forms. Take 5-mdC as an example, the percentage of 5-mdC was calculated using following equations: 5-mdC% = 100 × Q_5-mdC_/(Q_dC_ + Q_5-mdC_ + Q_5-hmdC_ + Q_5-fdC_ + Q_5-cadC_), and Q_dC_, Q_5-mdC_, Q_5-hmdC,_ Q_5-fdC_, Q_5-cadC_ values are the molar quantities of dC, 5-mdC, 5-hmdC, 5-fdC and 5-cadC, respectively.

### Global histone modifications

#### Histone protein extraction

One hundred micrograms of tissue were added 1,000 µl of Triton extraction buffer (TEB included PBS containing 0.5% Triton X-100, 5 mM sodium butyrate (NaBu) and Protease inhibitors) and use four beads of 2.8 mm ceramic beads and homogenization with bead beater (following the protocol liver: 5,000 rpm, 2 × 10s, 15 s break, muscle: 5,500 rpm, 2 × 20s, 15 s break). After that, samples were incubated on ice for 20 min. The homogenised mixture was transferred to a new tube and centrifuged at 2,000 rpm, 4°C, for 10 min. Thereafter, the supernatants were discarded, and the pellet was resuspended in acid extraction buffer (AEB, which included 0.5 N HCl and 10% glycerol). Then, samples were incubated on ice for 30 min and vortexed briefly every 10 min. After incubation, the samples were centrifuged at 12,000 rpm for 5 min at 4°C. Next, the supernatant was transferred to a new tube, and subsequently, three volumes of cold acetone were added for protein precipitation at −20°C overnight. After protein precipitation, the protein pellets were collected via centrifugation at 12,000 rpm for 5 min at 4°C, and the samples were aspirated. Protein samples were washed with 1 ml of cold acetone, and the protein pellets were dried on ice. Protein samples were dissolved in distilled water and then warmed at 45–60°C for 1 h to dissolve the pellets completely. The samples were aliquoted for protein quantification and stored at −80°C for further use.

#### Sample preparation

Samples were prepared with 5 µg of protein and mixed with 1X loading solution, together with 2-mercaptoethanol (BME). Samples were mixed and heated on a dry bath at 95–98°C for 5 min. Then, the samples were aliquoted and kept at −20°C for western blotting assays.

#### Western blotting and histone antibodies

Five micrograms of total protein were subjected to SDS-PAGE and western blotting using the specific antibodies on 15% gel (40% acrylamide, 2% bis-acrylamide, 1.5 M Tris-HCl pH 8.8, 10% SDS) for 100 min at 100 V and then 75 min at 100 mA, according to the publication of Marandel et al. [2]. Specific histone antibodies were used for Western blotting analysis. Prior to analysis, appropriate amounts of protein samples from muscle and liver, as well as antibody dilutions, were optimised and validated. Primary antibodies were used at a 1:1,000 dilution in Intercept™ (PBS) Blocking Buffer (LICORbio, USA; cat. no. 927–70001): anti-H3K4me3 (polyclonal; Diagenode, Belgium; cat. no. C15410003), anti-H3K9me3 (polyclonal; Diagenode, Belgium; cat. no. C15410056), anti-H3K9ac (polyclonal; Diagenode, Belgium; cat. no. C15410177), and anti-H3 (polyclonal; Abcam, UK; cat. no. ab1791). The secondary antibody, goat anti-rabbit IgG H&L (HRP) (polyclonal; Abcam, UK; cat. no. ab205718), was used at a 1:10,000 dilution in the same blocking buffer.

### Statistical analysis

The statistical model used was y_ij_ = µ + α_i_ + ε_ij_, where y_ij_ was the response, µ was the general mean, α_i_ was the effect of nutritive status (fed and fasted, and LC/HP and HC/LP refed), and ε_ij_ was the random error. All data were analysed using SPSS for Windows, version 22 (SPSS Inc., Chicago, IL, USA). Normality of distributions was assessed using the Shapiro–Wilk test. Data were analysed using a Kruskal – Wallis non-parametric test, followed by a Tukey test as a post hoc analysis when data did not follow a normal distribution. In addition, if the data followed a normal distribution, a one-way analysis of variance (ANOVA) was performed. When significant differences were observed, Tukey’s range test was performed to rank the treatment groups. Effects and differences were considered significant at *p* < 0.05.

## Results

### Effects of short-term refeeding with dietary low- or high-CHO on global DNA methylation landscape in juvenile and adult Nile tilapia

**Figure 2.**
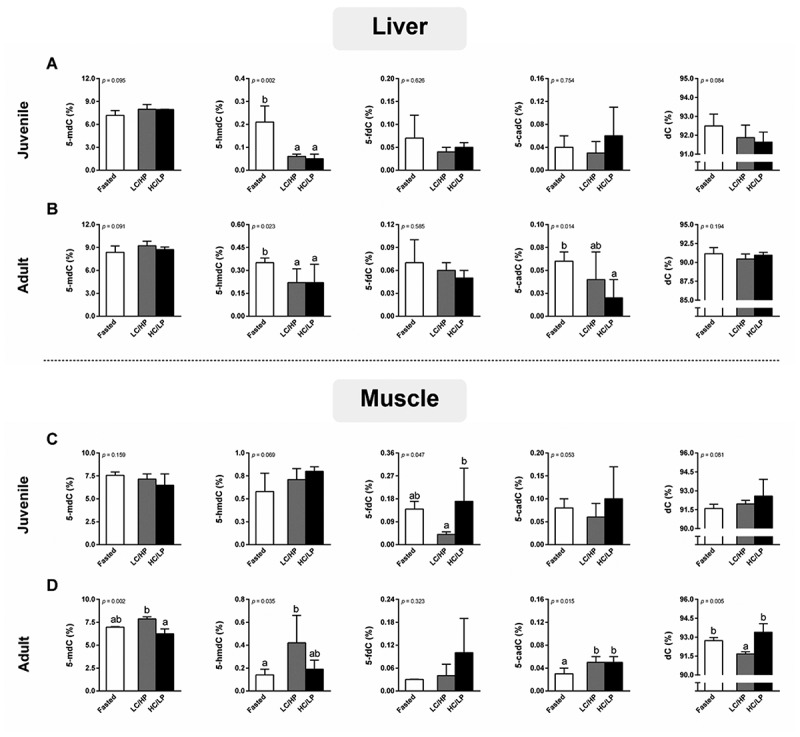
Global DNA methylation and their demethylation derivatives concentrations in liver of juvenile (A) and adult (B), and in muscle of juvenile (C) and adult (D) Nile tilapia. Fish were subjected to fasting for 4 days fast (fasted), followed by 4 days of refeeding (refed) with either a low-carbohydrate/high-protein (LC/HP) or high-carbohydrate/low-protein (HC/LP) diets. Data represent means ± standard deviation (SD; *n* = 6 individuals per experimental group). Different lowercase letters indicate significant differences among nutritional status (*p* < 0.05). 5-mdC, 5-methyl-2’ -deoxycytidine; 5-hmdC, 5-hydroxymethyl-2’ -deoxycytidine; 5-fdC, 5-formyl-2’ -deoxycytidine; 5-cadC, 5-carboethoxy-2’ -deoxycytidine; dC, 2’-deoxycytidine.

Figure 2 shows the modification of global DNA (de)methylation derivatives including 5-mdC, 5-hmdC, 5-fdC, 5-cadC, and dC in the liver and muscle of experimental fish. In juvenile fish, irrespective of dietary refeeding, decreased hepatic global 5-hmdC content compared to fasted fish (*p* < 0.05). However, there were no significant differences in global 5-mdC, 5-fdC, 5-cadC, and dC in the liver of juveniles between fasted and refed states (*p* > 0.05) (Figure 2(A)). In muscle, although muscular 5-mdC content between fasted and refed states in juveniles remains unchanged, the elevation of muscular 5-fdC derivative content was observed in juvenile refed with dietary HC/LP compared to LC/HP groups (*p* < 0.05) (Figure 2(C)).

In adults, the modification of global DNA (de)methylation derivatives in the liver and muscle is illustrated in Figure 2(B,D), respectively. Compared to fasting, subsequently refeeding with either LC/HP or HC/LP diets decreased hepatic 5-hmdC content (*p* < 0.05), while a decrease of 5-cadC content was observed in only adult refed with HC/LP diet (*p* < 0.05). There were no significant differences in hepatic 5-mdC, 5-fdC, and dC contents between fasted and refed adult Nile tilapia (*p* > 0.05) (Figure 2(B)). In adult muscle, compared to fasted, although 5-mdC content was not affected by short-term refeeding (*p* > 0.05), a higher muscular 5-cadC content was observed in adult refed with either LC/HP or HC/LP diets (*p* < 0.05) (Figure 2(D)). Compared to fasted, dietary LC/HP increased muscular 5-hmdC and decreased dC contents (*p* < 0.05), while the muscular (de)methylation derivatives (except 5-cadC) between fasted and HC/LP refeeding groups remain unchanged (*p* > 0.05). Between refed diets, dietary HC/LP decreased in muscular 5-mdC, while dC contents increased compared to adults refed with an LC/HP diet (*p* < 0.05). There were no significant differences in 5-hmdC, 5-fdC, and 5-cadC contents between LC/HP and HC/LP diets (*p* > 0.05).

### Effects of short-term refeeding with dietary low- or high-CHO on expression of genes related to DNA (de)methylation in juvenile and adult Nile tilapia

This study evaluated hepatic mRNA levels of genes related to DNA (de)methylation in fasted juvenile and adult Nile tilapia (Table 3). Juveniles, irrespective of dietary refeeding, exhibited higher mRNA level of the hepatic *dnmt3bb* gene compared to fasted fish (*p* < 0.05). Except for *dnmt1* in LC/HP, short-term refeeding with the HC/LP diet showed the highest expression levels of hepatic DNA methylation writers (*dnmt1*, *dnmt3aa*, and *dnmt3ba*) compared to the fasted group (*p* < 0.05). For the DNA methylation eraser, the mRNA levels of the *tet1* and *tet2* genes were higher in fasted juveniles compared to refed fish (*p* < 0.05). Moreover, the mRNA level of *tet3* was significantly higher in fasted and HC/LP-refed fish compared with fish refed with the LC/HP diet (*p* < 0.05).

**Table 3.** mRNA levels of genes involved in DNA (de)methylation in the liver of juvenile and adult Nile tilapia fasted for 4 days and refed for 4 days with either an LC/HP or an HC/LP diet (mean ± standard deviation [SD], *n* = 6).

Genes involved in Epigenetics modification		Juvenile stage				Adult stage			
		Fasted	LC/HP	HC/LP	*p*-value	Fasted	LC/HP	HC/LP	*p*-value
DNA methylation writers	*dnmt1*	0.40 ± 0.00^a^	0.98 ± 0.03^ab^	1.75 ± 0.09^b^	0.001	1.78 ± 0.38^b^	0.30 ± 0.02^a^	1.31 ± 0.11^ab^	0.002
	*dnmt3aa*	0.52 ± 0.05^a^	0.62 ± 0.10^a^	0.84 ± 0.08^b^	<0.001	1.31 ± 0.16^b^	0.38 ± 0.04^a^	0.69 ± 0.03^ab^	0.001
	*dnmt3ab*	nd	nd	nd		nd	nd	nd	
	*dnmt3ba*	0.22 ± 0.06^a^	0.13 ± 0.01^a^	0.67 ± 0.12^b^	0.001	0.32 ± 0.05^a^	0.55 ± 0.08^b^	0.89 ± 0.16^c^	<0.001
	*dnmt3bb*	0.21 ± 0.02^a^	0.50 ± 0.08^b^	0.67 ± 0.12^b^	0.001	0.33 ± 0.05^a^	0.58 ± 0.04^ab^	0.90 ± 0.16^b^	0.001
DNA methylation erasers	*tet1*	0.69 ± 0.15^b^	0.35 ± 0.08^a^	0.43 ± 0.12^a^	0.001	1.16 ± 0.17^b^	0.21 ± 0.03^a^	0.31 ± 0.04^ab^	0.001
	*tet2*	0.70 ± 0.10^b^	0.42 ± 0.06^a^	0.52 ± 0.14^a^	0.001	1.58 ± 0.45^b^	0.30 ± 0.09^a^	0.70 ± 0.02^ab^	0.001
	*tet3*	0.77 ± 0.01^b^	0.50 ± 0.06^a^	0.67 ± 0.10^b^	<0.001	1.20 ± 0.15^c^	0.45 ± 0.13^a^	0.73 ± 0.18^b^	<0.001

Abbreviations: LC/HP = low carbohydrate and high protein; HC/LP = high carbohydrate and low protein.

Means with different superscripts in each row differ significantly (*p* < 0.05).

In adults, the regulation of genes related to DNA (de)methylation was affected by dietary LC/HP and HC/LP independently (Table 3). Compared to fasted, mRNA levels of *the dnmt1* and *dnmt3aa* genes were lower only in fish refed with the LC/HP diet (*p* < 0.05). However, the mRNA level of *dnmt3ba* was higher in refeeding fish, whereas the mRNA level of the *dnmt3bb* gene was significantly higher in HC/LP refeeding fish compared to fasted ones (*p* < 0.05). When examining the diets, the mRNA level of *dnmt3ba* was higher in HC/LP-fed fish compared to the LC/HP group (*p* < 0.05). For the hepatic DNA methylation eraser, the mRNA level of *tet3* in adults was higher in fasted fish compared to refed ones (*p* < 0.05). However, compared to fasted, adults refed with the LC/HP diet showed significantly lower mRNA levels of the *tet1* and *tet2* genes (*p* < 0.05). After feed deprivation, the mRNA level of the *tet3* gene was significantly higher in HC/LP refeeding compared to LC/HP fish (*p* < 0.05). No significant difference was found in the mRNA levels of *dnmt1, dnmt3aa, dnmt3bb, tet1*, and *tet2* genes between HC/LP and LC/HP refeeding groups (*p* > 0.05).

At the molecular level in muscle tissue (Table 4), juvenile tilapia refed with either LC/HP or HC/LP diets exhibited significantly lower mRNA levels of DNA methylation writers (*dnmt1* and *dnmt3ba*) and erasers (*tet2* and *tet3*) compared to fasted fish (*p* < 0.05). In contrast, refeeding resulted in significantly higher mRNA levels of *dnmt3aa* and *dnmt3bb* relative to the fasted group (*p* < 0.05). Between the two refeeding diets, juveniles refed with the HC/LP diet showed significantly higher mRNA levels of DNA methylation writer genes (*dnmt1*, *dnmt3aa*, and *dnmt3bb*) and the eraser gene *tet3* compared to those refed with LC/HP (*p* < 0.05). No significant differences in *tet1* expression were observed among the fasted, LC/HP, and HC/LP groups (*p* > 0.05).

**Table 4.** mRNA levels of gene involved in DNA (de)methylation in muscle of juvenile and adult Nile tilapia that were fasted 4 days and refed 4 days with either an LC/HP or an HC/LP diet (mean ± standard deviation [SD], *n* = 6).

Genes involved in Epigenetics modification		Juvenile stage				Adult stage			
		Fasted	LC/HP	HC/LP	*p*-value	Fasted	LC/HP	HC/LP	*p*-value
DNA methylation writers	*dnmt1*	2.11 ± 0.12^c^	1.44 ± 0.08^a^	1.75 ± 0.06^b^	<0.001	1.01 ± 0.07^a^	1.21 ± 0.05^ab^	1.82 ± 0.28^b^	0.001
	*dnmt3aa*	0.84 ± 0.03^a^	1.29 ± 0.04^b^	1.54 ± 0.03^c^	<0.001	0.95 ± 0.02^a^	1.39 ± 0.02^c^	0.99 ± 0.04^b^	<0.001
	*dnmt3ab*	nd	nd	nd		nd	nd	nd	
	*dnmt3ba*	1.87 ± 0.08^b^	1.05 ± 0.12^a^	1.17 ± 0.22^a^	<0.001	1.13 ± 0.19^b^	0.84 ± 0.13^a^	1.12 ± 0.11^b^	0.006
	*dnmt3bb*	0.83 ± 0.10^a^	1.45 ± 0.04^b^	1.79 ± 0.12^c^	<0.001	0.60 ± 0.06^a^	0.82 ± 0.03^b^	1.28 ± 0.07^c^	<0.001
DNA methylation erasers	*tet1*	1.39 ± 0.07	1.38 ± 0.05	1.45 ± 0.12	0.272	1.05 ± 0.01^a^	1.00 ± 0.04^a^	1.22 ± 0.04^b^	<0.001
	*tet2*	1.59 ± 0.08^b^	0.86 ± 0.02^a^	0.98 ± 0.06^a^	0.001	1.33 ± 0.04^b^	0.76 ± 0.04^a^	1.04 ± 0.09^b^	0.001
	*tet3*	1.46 ± 0.05^c^	0.91 ± 0.02^a^	1.15 ± 0.03^b^	<0.001	1.08 ± 0.05^b^	0.76 ± 0.04^a^	1.26 ± 0.13^b^	0.001

Abbreviations: LC/HP = low carbohydrate and high protein; HC/LP = high carbohydrate and low protein.

Means with different superscripts in each row differ significantly (*p* < 0.05).

In adults, the alteration of genes related to muscular DNA (de)methylation was affected by dietary LC/HP and HC/LP independently (Table 4). Compared to the fasted state, mRNA levels of DNA methylation writer genes (*dnmt3aa* and *dnmt3bb*) were higher in fish refed with either LC/HP or HC/LP diets (*p* < 0.05). However, compared to fasted adults, those refed with the LC/HP diet showed significantly lower mRNA levels of *dnmt3ba, tet2*, and *tet3*. In contrast, the mRNA levels of *dnmt1* and *tet1* were significantly higher in adults refed with the HC/LP diet (*p* < 0.05). Between refeeding diets, the lower mRNA level of *dnmt3aa* and, together with higher mRNA levels of *dnmt3ba, dnmt33bb, tet1, tet2*, and *tet3* genes, were found in adult refed with HC/LP compared to the LC/HP refeeding group (*p* < 0.05).

### Effects of short-term refeeding with dietary low- or high-CHO on global histone modification landscape in juvenile and adult Nile tilapia

**Figure 3.**
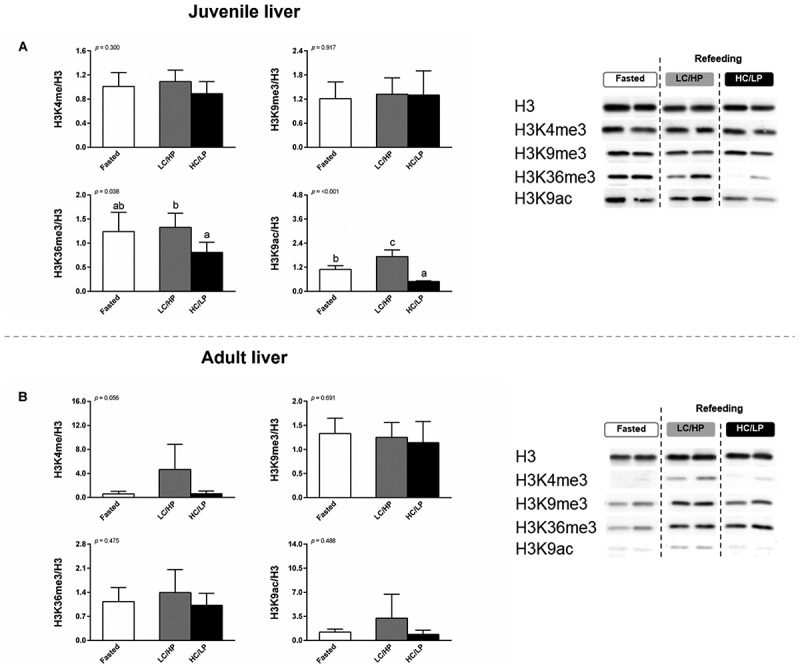
Global histone modification in liver of juvenile (A) and adult (B) Nile tilapia. Fish were subjected to fasting for 4 days (fasted), followed by refeeding for 4-days (refed) with either a low-carbohydrate/high-protein (LC/HP) or high-carbohydrate/low-protein (HC/LP) diets. Data represent means ± standard deviation (SD; *n* = 6 individuals per experimental group). Different lowercase letters indicate significant differences among nutritional status (*p* < 0.05).

**Figure 4.**
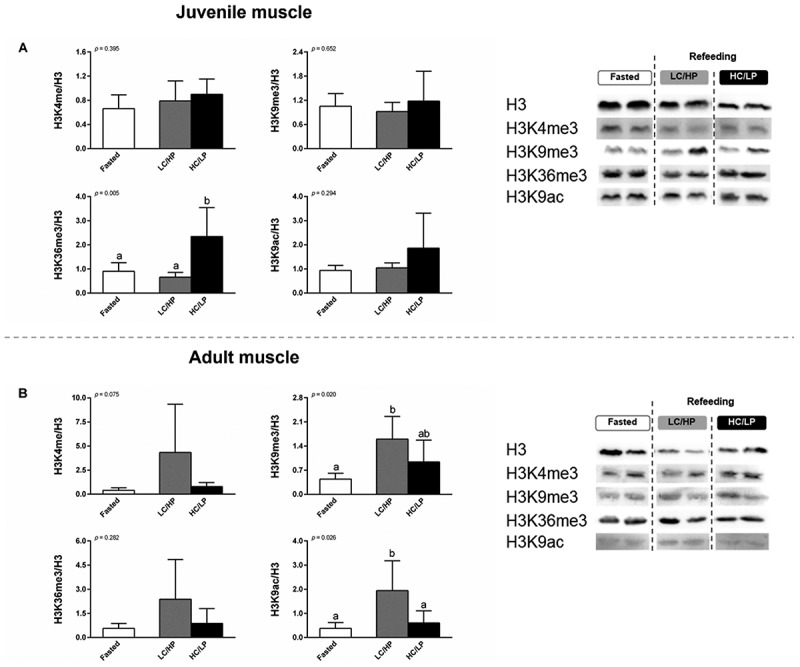
Global histone modification in muscle of juvenile (A) and adult (B) Nile tilapia. Fish were subjected to fasting for 4 days (fasted), followed by refeeding for 4 days (refed) with either a low-carbohydrate/high-protein (LC/HP) or high-carbohydrate/low-protein (HC/LP) diets. Data represent means ± standard deviation (SD; *n* = 6 individuals per experimental group). Different lowercase letters indicate significant differences among nutritional status (*p* < 0.05).

Global levels of selected histone modifications (H3K4me3, H3K9me3, H3K36me3, and H3K9ac) were measured in both liver and muscle of Nile tilapia (Figures 3 and 4). In the liver, compared to fasted, a higher and lower enrichment of global hepatic H3K9ac was detected in juveniles refed with LC/HP and HC/LP diets, respectively (*p* < 0.05) (Figure 3(A)). Between dietary refeeding conditions, a lower enrichment of global H3K36me3 and H3K9ac was exhibited in the liver of juveniles refed with HC/LP diet (*p* < 0.05). There were no significant differences in H3K4me3 and H3K9me3 in juvenile Nile tilapia among experimental conditions (*p* > 0.05). In adults, the global levels of hepatic H3K4me3, H3K9me3, H3K36me3, and H3K9ac remained stable between fasted and refed conditions (*p* > 0.05) (Figure 3(B)).

Compared to fasting, refeeding with either LC/HP and/or HC/LP modulated several histone marks (Figure 4). In juveniles, short-term refeeding with dietary HC/LP resulted in higher global enrichment of muscular H3K36me3 compared to fasted and refed groups with LC/HP (*p* < 0.05). There were no significant differences in muscular H3K4me3, H3K9me3, and H3K9ac in juveniles between fasted and refed groups (*p* > 0.05) (Figure 4(A)). In adults, compared to the fasted state, higher enrichment in muscular H3K9me3 and H3K9ac was exhibited in the LC/HP refeeding group (*p* < 0.05). Indeed, global enrichment of muscular H3K9ac was increased in adults refed with dietary LC/HP compared to the HC/LP diet (*p* < 0.05). There were no significant differences in muscular H3K4me3 and H3K36me3 in adults between fasted and refed conditions (*p* > 0.05) (Figure 4(B)).

### Effects of short-term refeeding with dietary low- or high-CHO on expression of genes related to histone modification in juvenile and adult Nile tilapia

After fasting, subsequent refeeding affects hepatic mRNA levels of genes related to histone modification in juveniles and adults independently (Table 5). In juvenile, fish refed with either LC/HP or HC/LP diets showed significantly lower mRNA levels of genes related to H3K4me3 writer (*setd1ba, kmt2a, kmt2ba*, and *kmt2bb*), H3K9ac writer (*kat6a*). In contrast, the higher mRNA levels of genes associated with H3K9me3 writer (*suv39h1b*), H3K9me3 and H3K36me3 eraser (*kdm4ab, kdm4c*), and H3K9ac writer (*kat2a, gtf3c4*) were observed in refeeding group when compared to fasted group (*p* < 0.05). Moreover, compared to fasted, the significantly lower mRNA levels of genes related to H3K4me3 (writer; *setd1a, kmt2bb*, and eraser; *kdm5ba*), H3K9me3 and H3K36me3 eraser (*kdm4aa*) in LC/HP refeeding group and significantly higher mRNA levels of genes related to H3K4me3 eraser (*kdm5a, kdm5ba, kdm5bb, kdm5c*, *riox1*), H3K9me3 and H3K36me3 eraser (*kdm4aa*, *kdm4b*), H3K9ac eraser (*sirt2, sirt5*) in HC/LP refeeding group were found in juvenile stage (*p* < 0.05). Between refeeding diets, the mRNA levels of genes related to H3K4me3 (writer; *setd1a*, and eraser; *kdm5a, kdm5ba, kdm5bb, kdm5c*), H3K9me3 writer (*suv39h1b*), H3K9me3 and H3K36me3 eraser (*kdm4aa, kdm4ab, kdm4b, kdm4c*), and H3K9ac (writer; *kat2b*, and eraser; *sirt5*) were higher in juvenile refed with dietary HC/LP compared with LC/HP diet (*p* < 0.05).

**Table 5.** mRNA levels of gene involved in histone modifications in liver of juvenile and adult Nile tilapia that were fasted 4 days and refed 4 days with either an LC/HP or an HC/LP diet (mean ± standard deviation [SD], *n* = 6).

Genes involved in Epigenetics modification		Juvenile stage				Adult stage			
		Fasted	LC/HP	HC/LP	*p*-value	Fasted	LC/HP	HC/LP	*p*-value
H3K4me3 writers	*setd1a*	0.63 ± 0.03^b^	0.47 ± 0.04^a^	0.68 ± 0.07^b^	0.003	1.94 ± 0.46^b^	0.29 ± 0.11^a^	0.68 ± 0.22^a^	0.001
	*setd1ba*	0.46 ± 0.03^b^	0.38 ± 0.07^a^	0.35 ± 0.05^a^	0.014	2.21 ± 0.40^c^	0.35 ± 0.14^a^	0.88 ± 0.31^b^	<0.001
	*kmt2a*	1.01 ± 0.10^b^	0.39 ± 0.08^a^	0.48 ± 0.05^a^	<0.001	1.52 ± 0.17^b^	0.64 ± 0.21^a^	0.88 ± 0.10^a^	<0.001
	*kmt2ba*	0.96 ± 0.02^b^	0.57 ± 0.02^a^	0.57 ± 0.10^a^	0.009	1.80 ± 0.25^b^	0.55 ± 0.25^a^	0.81 ± 0.16^a^	<0.001
	*kmt2bb*	0.84 ± 0.09^b^	0.67 ± 0.09^a^	0.75 ± 0.04^ab^	0.010	2.06 ± 0.36^c^	0.53 ± 0.20^a^	0.94 ± 0.11^b^	<0.001
H3K4me3 erasers	*kdm5a*	0.46 ± 0.07^a^	0.51 ± 0.02^a^	1.18 ± 0.12^b^	0.003	1.89 ± 0.07^c^	0.18 ± 0.05^a^	0.64 ± 0.12^b^	<0.001
	*kdm5ba*	0.82 ± 0.08^b^	0.64 ± 0.10^a^	1.32 ± 0.10^c^	<0.001	2.71 ± 0.99^b^	0.36 ± 0.06^a^	1.02 ± 0.22^a^	0.001
	*kdm5bb*	0.34 ± 0.05^a^	0.25 ± 0.04^a^	0.64 ± 0.11^b^	<0.001	1.03 ± 0.29^b^	0.11 ± 0.05^a^	0.38 ± 0.09^ab^	0.001
	*kdm5c*	0.30 ± 0.04^a^	0.25 ± 0.06^a^	0.42 ± 0.07^b^	0.001	0.81 ± 0.32^b^	0.14 ± 0.06^a^	0.35 ± 0.08^ab^	0.001
	*riox1*	0.09 ± 0.04^a^	0.29 ± 0.09 ^ab^	0.84 ± 0.38^b^	0.001	0.80 ± 0.33^b^	0.07 ± 0.05^a^	0.42 ± 0.28^b^	0.002
H3K9me3 specific writer	*suv39h1b*	0.19 ± 0.04^a^	0.67 ± 0.06^b^	0.96 ± 0.06^c^	<0.001	1.01 ± 0.28^b^	0.24 ± 0.07^a^	0.41 ± 0.11^ab^	0.001
H3K9me3 and H3K36me3 erasers	*kdm4aa*	0.45 ± 0.02^b^	0.36 ± 0.03^a^	0.53 ± 0.02^c^	<0.001	1.90 ± 0.13^c^	0.25 ± 0.04^a^	0.73 ± 0.06^b^	<0.001
	*kdm4ab*	0.25 ± 0.02^a^	0.32 ± 0.05^b^	0.49 ± 0.03^c^	<0.001	1.10 ± 0.06^b^	0.17 ± 0.01^a^	0.51 ± 0.02^ab^	0.002
	*kdm4b*	0.35 ± 0.04^a^	0.37 ± 0.03^a^	0.75 ± 0.05^b^	<0.001	1.22 ± 0.08^b^	0.24 ± 0.05^a^	1.25 ± 0.09^b^	<0.001
	*kdm4c*	0.26 ± 0.02^a^	0.30 ± 0.02^b^	0.56 ± 0.04^c^	<0.001	0.84 ± 0.05^c^	0.18 ± 0.03^a^	0.59 ± 0.03^b^	<0.001
H3K36me3 specific writer	*setd2*	0.45 ± 0.04	0.40 ± 0.01	0.44 ± 0.05	0.097	1.43 ± 0.03^c^	0.29 ± 0.03^a^	0.44 ± 0.02^b^	<0.001
H3K9ac specific writers	*kat2a*	0.22 ± 0.05^a^	0.45 ± 0.03^b^	0.39 ± 0.09^b^	<0.001	0.88 ± 0.21^c^	0.27 ± 0.09^a^	0.58 ± 0.14^b^	<0.001
	*kat2b*	0.47 ± 0.10^ab^	0.33 ± 0.13^a^	0.58 ± 0.19^b^	0.034	1.24 ± 0.46^b^	0.37 ± 0.12^a^	0.62 ± 0.22^ab^	0.003
	*kat6a*	0.70 ± 0.04^b^	0.26 ± 0.02^a^	0.22 ± 0.11^a^	<0.001	1.04 ± 0.18^b^	0.28 ± 0.09^a^	0.38 ± 0.15^a^	<0.001
	*gtf3c4*	0.40 ± 0.03^a^	0.51 ± 0.05^b^	0.52 ± 0.10^b^	0.009	0.78 ± 0.21^b^	0.33 ± 0.17^a^	0.59 ± 0.20^b^	0.004
H3K9ac specific erasers	*sirt2*	0.17 ± 0.02^a^	0.33 ± 0.05^ab^	1.24 ± 0.46^b^	0.001	1.14 ± 0.04^c^	0.06 ± 0.02^a^	0.32 ± 0.03^b^	<0.001
	*sirt5*	0.44 ± 0.02^a^	0.34 ± 0.07^a^	0.62 ± 0.03^b^	0.002	1.03 ± 0.15^b^	0.12 ± 0.02^a^	0.35 ± 0.06^ab^	<0.001

Abbreviations: LC/HP = low carbohydrate and high protein; HC/LP = high carbohydrate and low protein.

Means with different superscripts in each row differ significantly (*p* < 0.05).

However, in adults, the mRNA levels of genes related to H3K4me3 (except *kdm5bb, kdm5c*, and *riox1* in HC/LP), H3K9me3 writer (except *suv39h1b* in HC/LP) and H3K36me3 eraser (except *kdm4ab* and *kdm4b* in HC/LP), H3K36me3 writer, and H3K9ac (except *kat2b, gtf3c4*, and *sirt5* in HC/LP) were lower in refed fish compared to fasted fish (*p* < 0.05). After four days of refeeding, the mRNA levels of genes related to H3K4me3 (writer; *setd1ba, kmt2bb*, and eraser; *kdm5a, riox1*), H3K9me3 and H3K36me3 eraser (*kdm4aa, kdm4b*, and *kdm4c*), H3K36me3 writer (*setd2*), and H3K9ac (writer; *kat2a, gtf3c4*, and eraser; *sirt2*) were significantly higher in HC/LP fish compared to LC/HP fish (*p* < 0.05). No significant differences were observed in the mRNA levels of *setd1a*, *kmt2a*, *kmt2ba*, *kdm5ba*, *kdm5bb*, *kdm5c*, *suv39h1b*, *kdm4ab*, *kat2b*, *kat6a*, and *sirt5* between HC/LP and LC/HP diets (*p* > 0.05).

In muscle, subsequent refeeding affects the muscular mRNA levels of genes related to histone modification in juveniles and adults differently (Table 6). In juvenile, compared to fasted, except the higher mRNA levels of *riox1* and *kat2a* genes, the lower mRNA levels of genes related to H3K4me3 (except *kdm5a* and *kdm5ba* in HC/LP), H3K9me3, H3K36me3, and H3K9ac were observed in fish refed with either HC/LP or LC/HP diets (*p* < 0.05). After refeeding, mRNA levels of genes related to H3K4me3 (writer; *setd1ba, kmt2ba*, and eraser; *kdm5ba, riox1*), H3K9me3 and H3K36me3 eraser (*kdm4aa*, and *kdm4b*), and H3K9ac writer (*kat2a, kat6a*, and *gtf3c4*) were significantly higher in fish refed with HC/LP compared to LC/HP fish (*p* < 0.05). However, the significant differences in mRNA levels of *setd1a, kmt2a, kmt2bb, kdm5a, kdm5bb, kdm5c, suv39h1b, kdm4c, setd2, kat2b, sirt2*, and *sirt5* genes between HC/LP and LC/HP were not observed (*p* > 0.05).

**Table 6.** mRNA levels of gene involved in histone modifications in muscle of juvenile and adult Nile tilapia that were fasted 4 days and refed 4 days with either an LC/HP or an HC/LP diet (mean ± standard deviation [SD], *n* = 6).

Genes involved in Epigenetics modification		Juvenile stage				Adult stage			
		Fasted	LC/HP	HC/LP	*p*-value	Fasted	LC/HP	HC/LP	*p*-value
H3K4me3 writers	*setd1a*	1.57 ± 0.09^b^	1.25 ± 0.07^a^	1.24 ± 0.05^a^	<0.001	1.16 ± 0.04	1.21 ± 0.04	1.21 ± 0.16	0.262
	*setd1ba*	1.78 ± 0.10^c^	1.38 ± 0.06^a^	1.57 ± 0.08^b^	<0.001	1.01 ± 0.03^a^	1.00 ± 0.05^a^	1.17 ± 0.06^b^	<0.001
	*kmt2a*	1.51 ± 0.04^b^	0.79 ± 0.08^a^	0.78 ± 0.05^a^	<0.001	0.98 ± 0.06^b^	0.73 ± 0.05^a^	0.94 ± 0.09^b^	<0.001
	*kmt2ba*	1.34 ± 0.05^c^	0.86 ± 0.03^a^	0.99 ± 0.03^b^	<0.001	0.98 ± 0.01^b^	0.82 ± 0.03^a^	0.96 ± 0.09^b^	0.005
	*kmt2bb*	1.88 ± 0.12^b^	1.13 ± 0.11^a^	1.19 ± 0.04^a^	<0.001	1.04 ± 0.06^a^	0.96 ± 0.08^a^	1.30 ± 0.11^b^	<0.001
H3K4me3 erasers	*kdm5a*	1.86 ± 0.30^b^	0.94 ± 0.04^a^	1.18 ± 0.07^ab^	0.001	1.36 ± 0.08^c^	1.02 ± 0.04^a^	1.14 ± 0.09^b^	<0.001
	*kdm5ba*	1.19 ± 0.07^b^	0.96 ± 0.03^a^	1.23 ± 0.06^b^	<0.001	0.72 ± 0.04^ab^	0.65 ± 0.05^a^	0.78 ± 0.09^b^	0.006
	*kdm5bb*	1.80 ± 0.16^b^	0.94 ± 0.09^a^	0.98 ± 0.02^a^	0.003	1.15 ± 0.05^b^	0.86 ± 0.03^a^	0.88 ± 0.03^a^	<0.001
	*kdm5c*	1.42 ± 0.05^b^	1.07 ± 0.13^a^	1.07 ± 0.03^a^	0.006	1.01 ± 0.15^b^	0.78 ± 0.15^a^	0.88 ± 0.03^ab^	0.020
	*riox1*	0.82 ± 0.02^a^	1.42 ± 0.06^b^	1.88 ± 0.08^c^	<0.001	0.85 ± 0.03^a^	0.92 ± 0.04^b^	1.25 ± 0.05^c^	<0.001
H3K9me3 specific writer	*suv39h1b*	1.66 ± 0.23^b^	0.82 ± 0.08^a^	1.01 ± 0.08^a^	0.001	0.71 ± 0.09^a^	1.07 ± 0.07^b^	1.71 ± 0.05^c^	<0.001
H3K9me3 and H3K36me3 erasers	*kdm4aa*	1.35 ± 0.06^c^	0.90 ± 0.05^a^	1.06 ± 0.04^b^	<0.001	1.09 ± 0.03^c^	0.65 ± 0.03^a^	0.90 ± 0.02^b^	<0.001
	*kdm4b*	1.22 ± 0.02^c^	0.93 ± 0.01^a^	0.97 ± 0.04^b^	<0.001	0.82 ± 0.03^a^	0.83 ± 0.04^a^	1.23 ± 0.02^b^	<0.001
	*kdm4c*	1.60 ± 0.09^b^	1.17 ± 0.04^a^	1.18 ± 0.03^a^	<0.001	0.98 ± 0.03	0.97 ± 0.06	1.08 ± 0.09	0.051
H3K36me3 specific writer	*setd2*	1.51 ± 0.08^b^	1.34 ± 0.05^a^	1.41 ± 0.04^a^	0.001	1.17 ± 0.05	1.17 ± 0.05	1.26 ± 0.09	0.185
H3K9ac specific writers	*kat2a*	0.75 ± 0.03^a^	1.45 ± 0.05^b^	1.72 ± 0.09^c^	<0.001	0.72 ± 0.01^a^	1.17 ± 0.02^b^	1.19 ± 0.01^b^	<0.001
	*kat2b*	1.87 ± 0.13^b^	0.67 ± 0.04^a^	0.68 ± 0.06^a^	0.003	1.35 ± 0.13^b^	0.58 ± 0.02^a^	0.95 ± 0.11^ab^	0.001
	*kat6a*	1.60 ± 0.11^c^	0.93 ± 0.05^a^	1.08 ± 0.04^b^	<0.001	1.21 ± 0.02^b^	0.73 ± 0.02^a^	0.95 ± 0.06^b^	0.001
	*gtf3c4*	1.60 ± 0.14^c^	0.96 ± 0.05^a^	1.11 ± 0.09^b^	<0.001	1.02 ± 0.05^b^	0.88 ± 0.06^a^	1.31 ± 0.07^c^	<0.001
H3K9ac specific erasers	*sirt2*	1.18 ± 0.04^b^	0.95 ± 0.03^a^	0.96 ± 0.10^a^	0.003	0.96 ± 0.02^b^	0.71 ± 0.03^a^	0.94 ± 0.04^b^	<0.001
	*sirt5*	1.39 ± 0.08^b^	0.93 ± 0.06^a^	0.99 ± 0.12^a^	<0.001	0.99 ± 0.04^c^	0.78 ± 0.03^a^	0.88 ± 0.05^b^	<0.001

Abbreviations: LC/HP = low carbohydrate and high protein; HC/LP = high carbohydrate and low protein.

Means with different superscripts in each row differ significantly (*p* < 0.05).

In adults, alterations in muscular mRNA levels of histone modification were affected by dietary HC/LP and LC/HP independently (Table 6). Compared to fasted, lower mRNA levels of *kdm5a, kdm5bb, kdm4aa*, and *sirt5* genes, together with higher mRNA levels of *riox1, suv39h1b*, and *kat2a* genes, were observed in refeeding fish (*p* < 0.05). Moreover, compared to fasted, mRNA levels of genes related to H3K4me3 (writer; *kmt2a, kmt2ba*, and eraser; *kdm5c*) and H3K9ac (writer; *kat2b, kat6a, gtf3c4*, and eraser; *sirt2*) were lower in LC/HP refeeding fish, whereas the significantly higher in mRNA levels of genes related to H3K4me3 writer (*setd1ba* and *kmt2bb*), H3K9me3 and H3K36me3 eraser (*kdm4b*), and H3K9ac writer (*gtf3c4*) were observed in only adult refed with HC/LP diet (*p* < 0.05). There were no significant differences in *setd1a, kdm5ba, kdm4c*, and *setd2* genes between fasted and refed conditions (*p* > 0.05). At the end of refeeding, higher muscular mRNA levels of genes related to H3K4me3 (writer; *setd1ba, kmt2a, kmt2ba, kmt2bb*, and eraser; *kdm5a, kdm5ba, riox1*), H3K9me3 writer (*suv39h1b*), H3K9me3 and H3K36me3 eraser (*kdm4aa, kdm4b*), and H3K9ac (writer; *kat6a, gtf3c4*, and eraser; *sirt2, sirt5*) were observed in adult refed with HC/LP compared to LC/HP diet (*p* < 0.05). However, there were no significant differences in the mRNA levels of *kdm5bb, kdm5c, kdm4c, setd2*, and *kat2b* between fish refed with HC/LP and LC/HP diets (*p* > 0.05).

## Discussion

As originally proposed by Waddington, epigenetics refers to the study of how environmental or behavioural factors can alter gene expression without changing the underlying DNA sequence [1]. Numerous studies have shown that nutritional factors – such as refeeding, CHO, and protein intake – can influence the epigenetic landscape through mechanisms like DNA (de)methylation and histone modifications in mammals [7,14], amphibians [4] and fish [2,3]. In rainbow trout, although it is a poor user of CHO as an energy source, both nutritional status and high dietary CHO intake were shown to affect epigenetic regulation, leading to changes such as global DNA hypomethylation, hypoacetylation of H3K9ac, and remodeling of epigenetic modulators [2,3]. Previous study showed that refeeding for 4 days following a 4-day fast induced changes in intermediary metabolism in both juvenile and adult stages of Nile tilapia [29]. Furthermore, the effects of short-term refeeding with HC/LP diet on intermediary metabolism resembled the responses observed with long-term intake of HC diet, including the induction of glycolysis and lipogenesis, along with the suppression of gluconeogenesis and amino acid catabolism [29], suggesting a strong responsiveness to dietary CHO regardless of life stage. The present study builds upon these findings by investigating how the epigenetic landscape in Nile tilapia responds to short-term dietary CHO refeeding following a fasting period. Specifically, this study examined DNA (de)methylation, histone modifications, and the expression of related epigenetic modulators at the molecular level in liver and muscle tissues across different developmental stages. The results were analysed in relation to the fish’s nutritional status and different refeeding diets, with comparisons made among fasted, LC/HP, and HC/LP groups.

### Short-term refeeding with different dietary carbohydrate levels influenced global DNA (de)methylation and DNA methylation modulators in Nile tilapia

The DNA methylation landscape and its oxidative derivatives are highly responsive to environmental changes, particularly nutritional status and dietary composition (e.g., refeeding, CHO, protein). For instance, in rats, a LP maternal diet during pregnancy led to DNA hypermethylation in the offspring’s liver [14]. In fish, refeeding – especially with HC diets – has been shown to induce hepatic global DNA hypomethylation in juvenile rainbow trout [2,3]. Previous work in Nile tilapia demonstrated a strong ability to utilise HC diets during short-term refeeding, as reflected in phenotypic responses (increased hepatic glycogen and triglyceride content) and molecular adjustments (suppressed hepatic amino acid catabolism) in both juvenile and adult stages [29]. Building on this, the present study investigated how short-term fasting and subsequent refeeding with varying CHO levels affect the cytosine methylation landscape – including its oxidative derivatives – in juvenile and adult Nile tilapia. We also examined the mRNA levels of key enzymes involved in DNA methylation and demethylation. These included the DNA methyltransferase (DNMT) family, which regulates both de novo methylation and maintenance methylation [34,35], and the Ten-eleven translocation (TET) family, which catalyses active DNA demethylation through the stepwise oxidation of 5-mC to 5-hmC, 5-fC, and 5-caC [17,36].

In the liver, refeeding after fasting resulted in a decrease in 5-mC oxidative derivatives – specifically, 5-hmdC in both juveniles and adults, and 5-cadC in adults. However, no significant differences in hepatic 5-mdC levels were observed across nutritional statuses. Similarly, Liu et al. [3] reported a reduction in 5-hmC following a four-day fasting and four-day refeeding period in trout. However, in that study, it was accompanied by a decrease in 5-mdC and an increase in dC, highlighting species-specific differences in the epigenetic response to nutritional changes. Additionally, our results suggest that the effects of refeeding varied slightly across different developmental stages. In juveniles, unlike in adults, a general reduction in *tet* mRNA expression following refeeding was consistent with the observed decrease in 5-mC oxidative derivatives. Despite stable 5-mdC and dC levels in both age groups, the expression of *dnmt* genes was responsive to both nutritional status and dietary composition, though the pattern varied by gene. Similar findings have been reported in trout [2,3], where the authors suggested that more complex regulatory mechanisms, including post-transcriptional or protein-level controls, may be involved.

In muscle tissue, unlike in the liver, changes in nutritional status induced alterations in DNA (de)methylation derivatives, and most of these effects were primarily influenced by the composition of the refeeding diet (except 5-cadC in adults). In adult fish, refeeding with the HC/LP diet led to DNA hypomethylation – as evidenced by a decrease in 5-mdC – compared to the LC/HP-fed group. This was accompanied by increased dC levels and decreased 5-hmdC. This pattern was specific to adults and represents the first report of DNA methylation dynamics in the muscle of Nile tilapia. It suggests that, as previously shown in trout [3], muscle tissue in tilapia undergoes active DNA demethylation in response to dietary formulation. The observed differences in DNA methylation remodelling between growth stages may reflect developmental plasticity and/or adaptability to dietary CHO utilisation. For instance, in rainbow trout, Callet et al. [28] demonstrated that broodstock had a greater capacity to utilise HC diets than juveniles-a difference attributed to growth stage-specific energy demands and gluconeogenic balance. Overall, our findings suggest that dietary composition during refeeding can influence epigenetic stability, with HC/LP diets promoting global DNA hypomethylation in adult muscle. At the molecular level, increased *tet1* mRNA expression in adults fed the HC/LP diet corresponded with decreased 5-mdC and 5-hmdC levels and increased dC, supporting its role in active demethylation. Other DNA methylation-related genes (writers and erasers) responded to nutritional status and/or dietary composition but did not exhibit a direct correlation with global methylation patterns in either juveniles or adults. As previously suggested, regulation may occur at the post-transcriptional or protein level. In conclusion, refeeding and dietary HC appear to affect epigenetic stability in tilapia muscle through global DNA (de)methylation and associated modulators. Further investigation into the enzymatic activity of these modulators is warranted. However, this will require distinguishing between DNA methylation-related proteins with high sequence similarity in order to define their specific roles and expression profiles.

### Short-term refeeding with different dietary carbohydrate levels influenced global histone modification and histone modulators in Nile tilapia

Within the epigenetic landscape, histone modifications serve as key regulatory mechanisms that alter chromatin structure and, consequently, influence gene expression. These modifications are responsive to environmental cues, particularly nutritional status and dietary composition. For example, in amphibians, fasting followed by refeeding induced histone modifications – such as hypomethylation of H3K9 and H3K36—in the intestines of African clawed frogs [4]. Similarly, in fish, Marandel et al. [2] explained that refeeding fasted juvenile rainbow trout resulted in hepatic hypermethylation of H3K9, whereas the acetylation of H3K9 was influenced by dietary CHO.

In this study, we examined four histone modifications previously associated with metabolic disorders and dysregulation of glucose metabolism: permissive marks (H3K4me3, H3K9ac, H3K36me3) and the repressive mark H3K9me3 [37]. Histone modifications are reversible and regulated by the dynamic balance between writer and eraser enzymes involved in methylation and acetylation [38], processes known to be influenced by diet and feeding status. Our findings showed that histone modifications were primarily affected by dietary composition in both liver and muscle tissues across juvenile and adult stages. In juveniles, hepatic hyperacetylation of H3K9 was observed in fish fed the LC/HP diet, while muscular hypermethylation of H3K36 occurred in those fed the HC/LP diet. However, compared to fasting, hepatic H3K9ac levels were reduced in HC/LP-fed juveniles. In adults, while hepatic histone marks were unaffected by dietary treatments, muscular hypermethylation and hyperacetylation of H3K9 were evident in fish refed with the LC/HP diet compared to those given the HC/LP diet. These results indicate that even short-term refeeding with differing nutritional compositions can significantly influence global histone acetylation and methylation patterns in Nile tilapia, in a life stage-dependent manner. Notably, Marandel et al. [2] reported that hepatic hypoacetylation of H3K9 was observed in juvenile rainbow trout refed with HC diet compared to those on no-CHO diet. Taken together, our study suggests that dietary nutrient composition during refeeding can alter the histone modification landscape in Nile tilapia, with HC diets tending to induce hypomethylation or hypoacetylation of histone marks, particularly in juvenile stages.

Regarding genes encoding histone modification writers and erasers, only a few showed expression patterns consistent with the observed changes in histone marks. These genes were mainly associated with the erasure of H3K36me3 and H3K9ac. Specifically, the expression profiles of hepatic *kdm4b* and *kdm4c* in juveniles, as well as muscular *kdm4c* in adults, were consistent with the observed decrease in H3K36me3 levels in the respective tissues and developmental stages. Additionally, the increased expression of *sirt2* and *sirt5* in the liver of juveniles and the muscle of adults fed the HC/LP diet aligned with the reduction in H3K9ac levels. These findings suggest that even short-term refeeding with different dietary formulations can modulate the expression of histone-modifying enzymes in Nile tilapia, potentially contributing to changes in histone marks at the protein level. However, while many of the genes analysed were influenced by nutritional status and/or dietary composition, their mRNA expression changes did not always correspond to observed alterations in histone modification levels. This highlights the likelihood that regulation also occurs at the post-transcriptional or protein activity level. Taken together, our findings suggest that both refeeding status and HC diet may influence histone modification dynamics in Nile tilapia by modulating histone methylation and acetylation regulators. Taken together, our findings suggest that both refeeding status and HC diet may influence histone modification dynamics in Nile tilapia by modulating histone methylation and acetylation regulators. Future studies assessing enzyme activity are needed to clarify these mechanisms, particularly given the potential for regulation at the protein level despite similar amino acid sequences among modulators.

In conclusion, the epigenetic landscape of Nile tilapia is sensitive to short-term fasting and refeeding with LC or HC diets, in both juvenile and adult stages. Refeeding following fasting led to a reduction in global hepatic 5-mC oxidative derivatives, and dietary composition during refeeding impacted histone methylation and acetylation patterns. Notably, a HC/LP refeeding diet induced DNA hypomethylation in adult muscle and influenced histone mark hypomethylation and hypoacetylation in Nile tilapia, highlighting the importance of dietary CHO/protein balance in shaping epigenetic responses during different life stages.

## Supplementary Material

Supplementary Table_S1.docx

## Data Availability

The authors confirm that the data supporting the findings of this study are available within the article [and/or] data depositary https://doi.org/10.7910/DVN/S0PAOF
