# Supplementary material for: Refeeding with different levels of dietary carbohydrate modulates epigenetic stability through global DNA (de)methylation and histone modifications in juvenile and adult Nile tilapia (Oreochromis niloticus)
Source: Epigenetics. 2025 Sep 29;20(1):2566514. doi: 10.1080/15592294.2025.2566514 (PMC12482430; doi:10.1080/15592294.2025.2566514)
Supplement: Supplementary Table_S1.docx [file KEPI_A_2566514_SM6286.docx]

**Supplementary information**

**Refeeding with different levels of dietary carbohydrate modulates epigenetic stability through global DNA (de)methylation and histone modifications in juvenile and adult Nile tilapia (*Oreochromis niloticus*)**

Sirijanya Thongchaitriwat^a^, Suksan Kumkhong^a^, Cécile Heraud^b^, Karine Dias^b^, Stephane Panserat^b^, Surintorn Boonanuntanasarn^a,^* and Lucie Marandel^b,^*

^a^School of Animal Technology and Innovation, Institute of Agricultural Technology, Suranaree University of Technology, Nakhon Ratchasima, Thailand 30000

^b^INRAE, Université de Pau et des Pays de l'Adour, E2S UPPA, NuMéA, Saint-Pée-sur-Nivelle, France 64310

*Corresponding author:

S. Boonanuntanasarn; surinton@sut.ac.th, Tel: +6644224371, Fax: +6644224150

L. Marandel; lucie.marandel@inrae.fr

# **Table S1. Nucleotide sequences of genes related to epigenetic modulators**

| **Genes** | **Information** | |
| --- | --- | --- |
| ***dnmt1*** | **ENSEMBL NO.** | ENSONIG00000001574 |
|  | **NCBI NO.** | XM_025906327.1 |
|  | **Position of CDS in NCBI** | 139-4671 |
|  | **Primer F (5’-3’)** | CTCACACTGCGCTGTCTTGT |
|  | **Primer R (5’-3’)** | ACAACGCTGAGAGAGCAAGC |
|  | **Liver tissue sequences** | GTGTACCTTTGGTGTCTTGCAGGCCGGTCAGTACGGTGTTGCGCAGACCC GGCGCAGGGCCATCATCCTGGCTGCTGCTCCTGGAGAGAAGCTGCCACGC TATCCTGAGCCTCTGCATGTGTTTGCCCCCAGAGCTTGCTCTCTCAGCGT TGT |
|  | **Muscle tissue sequences** | CTTTGGTGTCTTGCAGGCCGGTCAGTACGGTGTTGCGCAGACCCGGCGCA GGGCCATCATCCTGGCTGCTGCTCCTGGAGAGAAGCTGCCACGCTATCCT GAGCCTCTGCATGTGTTTGCCCCCAGAGCTTGCTCTCTCAGCGTTGT |
|  |  |  |
| ***dnmt3aa*** | **ENSEMBL NO.** | ENSONIG00000005542 |
|  | **NCBI NO.** | XM_005475084.4 |
|  | **Position of CDS in NCBI** | 357-2885 |
|  | **Primer F (5’-3’)** | CCAACAACCACGAGCAGGAA |
|  | **Primer R (5’-3’)** | TGCCGACAGTGATGGAGTCT |
|  | **Liver tissue sequences** | GTTGTATCCTCCAGTTGCAGCAGAGAAGAGGCAACCAATCAGAGTCCTCT CTTTATTCGACGGCATTGCCACAGGTCTTCTGGTGCTGAAAGATTTGGGC ATCCAGGTCGAAAAGTATGTTGCATCAGAAGTGTGTGAAGACTCCATCAC TGTCGGCA |
|  | **Muscle tissue sequences** | GTTGTATCCTCCAGTTGCAGCAGAGAAGAGGCAACCAATCAGAGTCCTCT CTTTATTCGACGGCATTGCCACAGGTCTTCTGGTGCTGAAAGATTTGGGC ATCCAGGTCGAAAAGTATGTTGCATCAGAAGTGTGTGAAGACTCCATCAC TGTCGGCA |
|  |  |  |
| ***dnmt3ab*** | **ENSEMBL NO.** | ENSONIG00000001050 |
|  | **NCBI NO.** | XM_005477258.3 |
|  | **Position of CDS in NCBI** | 280-3228 |
|  | **Primer F (5’-3’)** | GCCGCAGCTTAGAGGACATC |
|  | **Primer R (5’-3’)** | CACACATGAGCACCTCTCGTC |
|  | **Liver tissue sequences** | CCTGTGGAAGTCTGAATGTTAGCCTTGAGCACCCTCTTTTTGCTGGAGGA ATGTGTCAGAGTTGCAAGAACTGCTTCCTAGAATGTGCTTATCAATACGA CGACGATGGCTACCAGTCATACTGC |
|  | **Muscle tissue sequences** | GAAGTCTGAATGTTAGCCTTGAGCACCCTCTTTTTGCTGGAGGAATGTGT CAGAGTTGCAAGAACTGCTTCCTAGAATGTGCTTATCAATACGACGACGA TGGCTACCAGTCATACTGC |
|  |  |  |
| ***dnmt3ba*** | **ENSEMBL NO.** | ENSONIG00000016781 |
|  | **NCBI NO.** | XM_025901732.1 |
|  | **Position of CDS in NCBI** | 184-4581 |
|  | **Primer F (5’-3’)** | GCTGCTGCAGATGCTACTGT |
|  | **Primer R (5’-3’)** | TTGCGCTGTTGTTGGCAAAG |
|  | **Liver tissue sequences** | GTGTGAACATCCTGGTGGGCCCAGAAACCTTTGATAAGCTGAAAGATGTCG ATCCGTGGAGCTGCTACATGTGCAAGCCATCACAGTGTGAAGGAAACCTCA AACTCAGGGCAGACTGGAGTGTGAAGGTCCAAGACTTCTTTGCCAACAACA GCGCAA |
|  | **Muscle tissue sequences** | TGAACATCCTGGTGGGCCCAGAAACCTTTGATAAGCTGAAAGATGTCGATC CGTGGAGCTGCTACATGTGCAAGCCATCACAGTGTGAAGGAAACCTCAAAC TCAGGGCAGACTGGAGTGTGAAGGTCCAAGACTTCTTTGCCAACAACAGCG CAA |
|  |  |  |
| ***dnmt3bb*** | **ENSEMBL NO.** | ENSONIG00000014841 |
|  | **NCBI NO.** | XM_025901790.1 |
|  | **Position of CDS in NCBI** | 378-2843 |
|  | **Primer F (5’-3’)** | TGCAGGAGTTCTTCGCCAAC |
|  | **Primer R (5’-3’)** | TGCCACATACTGACCCACCT |
|  | **Liver tissue sequences** | GAGTTTGAGAAACCAAAGATTTACCCAGCAGTCCCTGCAGAGCAGAGACGA CCAATCAGAGTCCTGTCCCTGTTTGACGGCATTGCCACAGGCTACCTGGTC CTGAGAGATCTGGGTTTTAAGGTGGGTCAGTATGTGGCA |
|  | **Muscle tissue sequences** | GTTTGAGAAACCAAAGATTTACCCAGCAGTCCCTGCAGAGCAGAGACGACC AATCAGAGTCCTGTCCCTGTTTGACGGCATTGCCACAGGCTACCTGGTCCT GAGAGATCTGGGTTTTAAGGTGGGTCAGTATGTGGCA |
|  |  |  |
| ***tet1*** | **ENSEMBL NO.** | ENSONIG00000033407 |
|  | **NCBI NO.** | XM_025897345.1 |
|  | **Position of CDS in NCBI** | 300-6794 |
|  | **Primer F (5’-3’)** | CATCCAGTCCCAGCACAACC |
|  | **Primer R (5’-3’)** | CTCTATTTGGCGTGCGCTGA |
|  | **Liver tissue sequences** | ACAATCCTCTGAAAGGAGCGCCTGTGTCAGCTATCAAATACAACTACAAA TCCCAGCAGCCCCCCAGTCAGAAAACACCTGTCAGGAAGAAAACGAAAGC TACGCCTTCCAAGCCCAGAAAGAAGAAAACTGATGGAATAAATCAGCGCA CGCCAAATAGAGA |
|  | **Muscle tissue sequences** | AAAGGAGCGCCTGTGTCAGCTATCAAATACAACTACAAATCCCAGCAGCC CCCCAGTCAGAAAACACCTGTCAGGAAGAAAACGAAAGCTACGCCTTCCA AGCCCAGAAAGAAGAAAACTGATGGAATAAATCAGCGCACGCCAAATAGA GA |
|  |  |  |
| ***tet2*** | **ENSEMBL NO.** | ENSONIG00000028706 |
|  | **NCBI NO.** | XM_005457001.3 |
|  | **Position of CDS in NCBI** | 545-5947 |
|  | **Primer F (5’-3’)** | GCAGCTGCCAACAAGAATGC |
|  | **Primer R (5’-3’)** | TGTTGCTGCTGCTGATGGAC |
|  | **Liver tissue sequences** | TGGAAAGCTCCAATGATAAAGGAGAGAAGGCCAAATCAAAAGCTGGCACT TTTGAGAATTCTACTCAGAGCAATCCAATGGCAGGACTTAATCCAGGTGC TGTAGGAGCAACCCGTCAGTCATGTCAGCCACAACACCCCCTAGGGGTCC ATCAGCAGCAGCAACA |
|  | **Muscle tissue sequences** | CTCCAATGATAAAGGAGAGAAGGCCAAATCAAAAGCTGGCACTTTTGAGA ATTCTACTCAGAGCAATCCAATGGCAGGACTTAATCCAGGTGCTGTAGGA GCAACCCGTCAGTCATGTCAGCCACAACACCCCCTAGGGGTCCATCAGCA GCAGCAACA |
|  |  |  |
| ***tet3*** | **ENSEMBL NO.** | ENSONIG00000015843 |
|  | **NCBI NO.** | XM_019365521.2 |
|  | **Position of CDS in NCBI** | 3585-9461 |
|  | **Primer F (5’-3’)** | GCAAGCCAACCAACCAAACC |
|  | **Primer R (5’-3’)** | GATGTGTTGGCTCCGACCTG |
|  | **Liver tissue sequences** | ACCCAGGCCCCAGTTTATAGCCAATCCCGTCCTTCCTCTGCCTCCTCTGA GTCCTCCAACAGAGGCACGCCGGTCATCAAACAGGAGCCTATGGATGTGC CAGTCTATGAAGGCACGCTGCCGAACCAGGTCGGAGCCAACACATC |
|  | **Muscle tissue sequences** | GGCCCCAGTTTATAGCCAATCCCGTCCTTCCTCTGCCTCCTCTGAGTCCT CCAACAGAGGCACGCCGGTCATCAAACAGGAGCCTATGGATGTGCCAGTC TATGAAGGCACGCTGCCGAACCAGGTCGGAGCCAACACATC |
|  |  |  |
| ***setd1a*** | **ENSEMBL NO.** | ENSONIG00000009854 |
|  | **NCBI NO.** | XM_005468973.4 |
|  | **Position of CDS in NCBI** | 62-6679 |
|  | **Primer F (5’-3’)** | GGAACTCCGGTCTGGATGGT |
|  | **Primer R (5’-3’)** | CGAAGCTGCCCATCTGTGTT |
|  | **Liver tissue sequences** | TTGTTTTTGAAGAAGACTTATGAGAAGCTGCTGCAGGATGACCATAGCTC TGACTGGCTCAATGACACTCATTGGGTCAACCATACTATAACCAATTTGC CAAATCCTCGCCGCAAAAAGAAGAACACAGATGGGCAGCTTCG |
|  | **Muscle tissue sequences** | TTGTTTTTGAAGAAGACTTATGAGAAGCTGCTGCAGGATGACCATAGCTC TGACTGGCTCAATGACACTCATTGGGTCAACCATACTATAACCAATTTGC CAAATCCTCGCCGCAAAAAGAAGAACACAGATGGGCAGCTTCG |
|  |  |  |
| ***setd1ba*** | **ENSEMBL NO.** | ENSONIG00000013898 |
|  | **NCBI NO.** | XM_005470275.4 |
|  | **Position of CDS in NCBI** | 890-6325 |
|  | **Primer F (5’-3’)** | AAGACAGGGAGGCAGCAGAA |
|  | **Primer R (5’-3’)** | CCTCAGGACTGGGAGGTCTG |
|  | **Liver tissue sequences** | GACTGCAAAGATGCTGCGACCTCCTCATCTTCTTCATCCTCTACATCTTC ATCTTCTGAGGATGAGGAGGAGGGGGAAGAGGCAGAGGCTAAGCCTCCGA GCTCTCCTGCAGTACCAATCCCAGAGGTCAAGGAGGAGCACATACGCAGA CCTCCCAGTCCTGAGG |
|  | **Muscle tissue sequences** | GTTGACTGCAAAGATGCTGCGACCTCCTCATCTTCTTCATCCTCTACATC TTCATCTTCTGAGGATGAGGAGGAGGGGGAAGAGGCAGAGGCTAAGCCTC CGAGCTCTCCTGCAGTACCAATCCCAGAGGTCAAGGAGGAGCACATACGC AGACCTCCCAGTCCTGAGG |
|  |  |  |
| ***kmt2a*** | **ENSEMBL NO.** | ENSONIG00000006229 |
|  | **NCBI NO.** | XM_013274782.3 |
|  | **Position of CDS in NCBI** | 50-13726 |
|  | **Primer F (5’-3’)** | AGAGCAGGAAAGCCAACAGC |
|  | **Primer R (5’-3’)** | CACTGGGCGTAGTTGTGGTC |
|  | **Liver tissue sequences** | AGTCCTTTTTCATCCGGCAAATGGACCGAACCTTTCCATGGTTCAAAGTG  AAAGAGTCCAGATTCTGGGAAAGCCAAAAAGTCTCTGCCAACAGTGGGCT  CCTCCCCAACGCTGTGCTGCCGCCGTCCCTAGACCACAACTACGCCCAGT  G |
|  | **Muscle tissue sequences** | AGTCCTTTTTCATCCGGCAAATGGACCGAACCTTTCCATGGTTCAAAGTG  AAAGAGTCCAGATTCTGGGAAAGCCAAAAAGTCTCTGCCAACAGTGGGCT  CCTCCCCAACGCTGTGCTGCCGCCGTCCCTAGACCACAACTACGCCCAGT  G |
|  |  |  |
| ***kmt2ba*** | **ENSEMBL NO.** | ENSONIG00000019760 |
|  | **NCBI NO.** | XM_013275905.3 |
|  | **Position of CDS in NCBI** | 95-9511 |
|  | **Primer F (5’-3’)** | ACTCTGAGGGACCTGGAGGA |
|  | **Primer R (5’-3’)** | AGAGGAGGTGAAGCCGATCC |
|  | **Liver tissue sequences** | GGCCTCGGAGGCTCTCGTCAAGAAGCCGTTGCAGCTCCTCGCCATCAGAG AGCGACCCGTCCGTACCGATGACACTCCGCTCTGGCGGCACGGTCCACTC CCGATGTGCCCTATTCAGCTCTCCTCCAAGGGCATCGAACTTCGGATCGG CTTCACCTCCTCT |
|  | **Muscle tissue sequences** | CTCGGAGGCTCTCGTCAAGAAGCCGTTGCAGCTCCTCGCCATCAGAGAGC GACCCGTCCGTACCGATGACACTCCGCTCTGGCGGCACGGTCCACTCCCG ATGTGCCCTATTCAGCTCTCCTCCAAGGGCATCGAACTTCGGATCGGCTT CACCTCCTCATA |
|  |  |  |
| ***kmt2bb*** | **ENSEMBL NO.** | ENSONIG00000002752 |
|  | **NCBI NO.** | XM_013277028.3 |
|  | **Position of CDS in NCBI** | 76-11661 |
|  | **Primer F (5’-3’)** | GCTCCCGTCAGTGTGTCTTC |
|  | **Primer R (5’-3’)** | TCTGGCTCCAACCCAGTCAA |
|  | **Liver tissue sequences** | GGTCTACTGCTACAAACACCGACATCTCATCAGTGGCAGGATGATAACGG GTCAGGAATTTGAAGTAAACCGCAGAGTGTATGTTGATTTTGAGGGGATC AGTCTCCGCAGAAAGTTCTTGACTGGGTTGGAGCCAGA |
|  | **Muscle tissue sequences** | ACTGCTACAAACACCGACATCTCATCAGTGGCAGGATGATAACGGGTCAG GAATTTGAAGTAAACCGCAGAGTGTATGTTGATTTTGAGGGGATCAGTCT CCGCAGAAAGTTCTTGACTGGGTTGGAGCCAGA |
|  |  |  |
| ***kdm5a*** | **ENSEMBL NO.** | ENSONIG00000009761 |
|  | **NCBI NO.** | XM_005451728.4 |
|  | **Position of CDS in NCBI** | 1028-6253 |
|  | **Primer F (5’-3’)** | TCTGGCCACAGAGGAGTTGT |
|  | **Primer R (5’-3’)** | GTGACGTGGCTCTGCTGAAA |
|  | **Liver tissue sequences** | GGCGAAGCTGTCTGTGCTGAGCCAGCGTATGGTTGAGCAGGCTGCGAGGG AGAAAACGGAGAAGATCATCAACGCTGAGCTGCAGAAAGCCGCTGCCAAT CCTGACTTACAGGGTCATATCCAAACTTTCCAGCAGTCTGGTTTCAGCAG AGCCACGTCAC |
|  | **Muscle tissue sequences** | CCCTGGCGAAGCTGTCTGTGCTGAGCCAGCGTATGGTTGAGCAGGCTGCG AGGGAGAAAACGGAGAAGATCATCAACGCTGAGCTGCAGAAAGCCGCTGC CAATCCTGACTTACAGGGTCATATCCAAACTTTCCAGCAGTCTGGTTTCA GCAGAGCCACGTCAC |
|  |  |  |
| ***kdm5ba*** | **ENSEMBL NO.** | ENSONIG00000019223 |
|  | **NCBI NO.** | XM_003441348.2 |
|  | **Position of CDS in NCBI** | 541-5001 |
|  | **Primer F (5’-3’)** | TCTCAGAGCAGAGGGCATCC |
|  | **Primer R (5’-3’)** | GACCCGATGTCACACCTTGG |
|  | **Liver tissue sequences** | ACTGGAGGCCCTTGTCAGTGATGTCCAGAGCTGGAAGGAGACGGCCACAA AGACATTCCTATTAAAAAACTCCCCTTTCTCACTTCTAGAGGTCCTCTGC CCAAGGTGTGACATCGGGTC |
|  | **Muscle tissue sequences** | GACTGGAGGCCCTTGTCAGTGATGTCCAGAGCTGGAAGGAGACGGCCACA AAGACATTCCTATTAAAAAACTCCCCTTTCTCACTTCTAGAGGTCCTCTG CCCAAGGTGTGACATCGGGTC |
|  |  |  |
| ***kdm5bb*** | **ENSEMBL NO.** | ENSONIG00000016770 |
|  | **NCBI NO.** | XM_003439103.5 |
|  | **Position of CDS in NCBI** | 249-4889 |
|  | **Primer F (5’-3’)** | CATCCCTGCCTACCTCCCAA |
|  | **Primer R (5’-3’)** | AAGGCTCCAGGTGGACTTGA |
|  | **Liver tissue sequences** | ACCATCAGAAAAGCCCGAGAGTGGCTGCAAGAAGCTGAGGAGCTTCAGGT CAGTGGCTGCATCCCGATGGTGGACACCCTCTCTGACATGGTGCTACGAG GACAAGCCATTCAAGTCCACCTGGAGCCTT |
|  | **Muscle tissue sequences** | ACCATCAGAAAAGCCCGAGAGTGGCTGCAAGAAGCTGAGGAGCTTCAGGT CAGTGGCTGCATCCCGATGGTGGACACCCTCTCTGACATGGTGCTACGAG GACAAGCCATTCAAGTCCACCTGGAGCCTT |
|  |  |  |
| ***kdm5c*** | **ENSEMBL NO.** | ENSONIG00000016838 |
|  | **NCBI NO.** | XM_005448517.4 |
|  | **Position of CDS in NCBI** | 551-5311 |
|  | **Primer F (5’-3’)** | CTCTCCACCCTGGAGGCAAT |
|  | **Primer R (5’-3’)** | AGCTACCAGGCCCTCCAAAT |
|  | **Liver tissue sequences** | CTTATCCCAGTCACGTTGCCCAACATTTTGGCTCTACAGGGGTGCCTTAC TCGAGCTCGGGCATGGGTAACAGACTTGGAGGAAATTCAGAATGGGGAGC ATTACCCATGTATGGATGATTTGGAGGGCCTGGTAGCTA |
|  | **Muscle tissue sequences** | CAACTTATCCCAGTCACGTTGCCCAACATTTTGGCTCTACAGGGGTGCCT  TACTCGAGCTCGGGCATGGGTAACAGACTTGGAGGAAATTCAGAATGGGG  AGCATTACCCATGTATGGATGATTTGGAGGGCCTGGTAGCTA |
|  |  |  |
| ***riox1*** | **ENSEMBL NO.** | ENSONIG00000005898 |
|  | **NCBI NO.** | XM_005475002.4 |
|  | **Position of CDS in NCBI** | 96-1727 |
|  | **Primer F (5’-3’)** | CCACCTGGCACACAAGGATT |
|  | **Primer R (5’-3’)** | TCCGGCTTCTACCACCACAT |
|  | **Liver tissue sequences** | TGATGACATTGAAGCCTTTGTGGTTCAGCTGGAGGGGAAGAAACGCTGGC GAGTGTACAGCCCAAGATCAGACGATGAGGTCTTGCCTGTGCTTTCGAGT CCAAACTTCGACCAGGCAGACATTGGTAAGCCGATCCTAGATGTGGTGGT AGAAGCCGGA |
|  | **Muscle tissue sequences** | TTATGATGACATTGAAGCCTTTGTGGTTCAGCTGGAGGGGAAGAAACGCT GGCGAGTGTACAGCCCAAGATCAGACGATGAGGTCTTGCCTGTGCTTTCG AGTCCAAACTTCGACCAGGCAGACATTGGTAAGCCGATCCTAGATGTGGT GGTAGAAGCCGGA |
|  |  |  |
| ***suv39h1b*** | **ENSEMBL NO.** | ENSONIG00000011649 |
|  | **NCBI NO.** | XM_003459875.5 |
|  | **Position of CDS in NCBI** | 87-1322 |
|  | **Primer F (5’-3’)** | TCCAACGCATGGCCTACAAC |
|  | **Primer R (5’-3’)** | CTTGATGTGCTGCAGTGTGC |
|  | **Liver tissue sequences** | GGTCCGGATTCGGCCTGGACAGCCAATATATGAGTGCAACTCCCGCTGCA GCTGTGGGCCTGACTGTCCCAACAGAGTTGTGCAGAATGGAATCCAGTTC GATCTGTGTATCTTTAAAACGGAGAATGGCCGGGGATGGGGAGTCCGCAC ACTGCAGCACATCAAG |
|  | **Muscle tissue sequences** | GGATTCGGCCTGGACAGCCAATATATGAGTGCAACTCCCGCTGCAGCTGT GGGCCTGACTGTCCCAACAGAGTTGTGCAGAATGGAATCCAGTTCGATCT GTGTATCTTTAAAACGGAGAATGGCCGGGGATGGGGAGTCCGCACACTGC AGCACATCAAG |
|  |  |  |
| ***kdm4aa*** | **ENSEMBL NO.** | ENSONIG00000007297 |
|  | **NCBI NO.** | XM_005457300.3 |
|  | **Position of CDS in NCBI** | 179-3472 |
|  | **Primer F (5’-3’)** | CGGATGCGAACCAAACCTCT |
|  | **Primer R (5’-3’)** | GGCTGGATCGACACCGTAAC |
|  | **Liver tissue sequences** | GTGTTTCACCACCACCACAGAGGACGACGCTGAGTGTGAAGAGCAACCGG TCCCTCCTCGTCTGGAAGAAGATGGAACCAGTCTCCTCATCAGCTGCTCA CAGTGCAGCGTGAGAGTTCATGCATCCTGTTACGGTGTCGATCCAGCC |
|  | **Muscle tissue sequences** | TGTGTTTCACCACCACCACAGAGGACGACGCTGAGTGTGAAGAGCAACCG GTCCCTCCTCGTCTGGAAGAAGATGGAACCAGTCTCCTCATCAGCTGCTC  ACAGTGCAGCGTGAGAGTTCATGCATCCTGTTACGGTGTCGATCCAGCC |
|  |  |  |
| ***kdm4ab*** | **ENSEMBL NO.** | ENSONIG00000010525 |
|  | **NCBI NO.** | XM_005476068.4 |
|  | **Position of CDS in NCBI** | 208-3738 |
|  | **Primer F (5’-3’)** | TCTGTTCAGGGAGGCACACA |
|  | **Primer R (5’-3’)** | GCCTGTTGGCCCATCTGTTT |
|  | **Liver tissue sequences** | ATTTATACGTAGATGTGCAGGTCATCCAAGAGGAGAAAGAGGAGTGGGCA AAGCCGCTAACCCAGTTATGGCAGTGTCTGCCATTTAACCCTGATGCAGA GAGGGAATATAACAAACAGATGGGCCAACAGGC |
|  | **Muscle tissue sequences** | TCCAAGAGGAGAAAGAGGAGTGGGCAAAGCCGCTAACCCAGTTATGGCAG TGTCTGCCATTTAACCCTGATGCAGAGAGGGAATATAACAAACAGATGGG CCAACAGGC |
|  |  |  |
| ***kdm4b*** | **ENSEMBL NO.** | ENSONIG00000012580 |
|  | **NCBI NO.** | XM_005453970.4 |
|  | **Position of CDS in NCBI** | 184-3897 |
|  | **Primer F (5’-3’)** | TGCTCGCTCTTCTGTCCGTA |
|  | **Primer R (5’-3’)** | AGCAGATCAGGAGGCTGGTT |
|  | **Liver tissue sequences** | TCCGCTGATAACGAAACCATGGGCGCTTCATTTCCCCGTCATGGCAGCTT GACACGTCCACTGGTCCCAGAGATGTGCTTCAGCGTGGGAGCAGGAAACA CGGAGCCACCGCCCACCAACTATCACATTGGGGAGGATGGAACCAGCCTC CTGATCTGCT |
|  | **Muscle tissue sequences** | GTCTCCGCTGATAACGAAACCATGGGCGCTTCATTTCCCCGTCATGGCAG CTTGACACGTCCACTGGTCCCAGAGATGTGCTTCAGCGTGGGAGCAGGAA ACACGGAGCCACCGCCCACCAACTATCACATTGGGGAGGATGGAACCAGC CTCCTGATCTGCT |
|  |  |  |
| ***kdm4c*** | **ENSEMBL NO.** | ENSONIG00000008824 |
|  | **NCBI NO.** | XM_005456806.2 |
|  | **Position of CDS in NCBI** | 231-5135 |
|  | **Primer F (5’-3’)** | CCTGCAGAGGAATGCAGTGG |
|  | **Primer R (5’-3’)** | GCACAGGTGCAATCTGGTGA |
|  | **Liver tissue sequences** | GGAGCCTGCAGATAAGGTTGGAATTTGCCCTTTGCCACCTGTACTGACCC AAGAGATGCCCTCGCTAACGCCTGCTGATGATGGCCTGACTGATTTTTCA AAATCTCGATCGTGTAGTCACCAGATTGCACCTGTGC |
|  | **Muscle tissue sequences** | CTCCGGAGCCTGCAGATAAGGTTGGAATTTGCCCTTTGCCACCTGTACTG ACCCAAGAGATGCCCTCGCTAACGCCTGCTGATGATGGCCTGACTGATTT TTCAAAATCTCGATCGTGTAGTCACCAGATTGCACCTGTGC |
|  |  |  |
| ***setd2*** | **ENSEMBL NO.** | ENSONIG00000003077 |
|  | **NCBI NO.** | XM_019364854.2 |
|  | **Position of CDS in NCBI** | 351-8159 |
|  | **Primer F (5’-3’)** | AGGCAGCGATGACTTCAAGC |
|  | **Primer R (5’-3’)** | ATCTTGTGGCGTCCCACTCT |
|  | **Liver tissue sequences** | AAGAGGTGAAGGAAAAACCTGATTTGGTTGTGGAAGAGAAGGACTCGGAT ATGAAAGAAGTCAAAAGTGAAGATCAGGCAGATGAGCTGGAACCACCAAA AGAGCCAGTTGAAACACAAGAGAGTGGGACGCCACAAGAT |
|  | **Muscle tissue sequences** | ACCTCCAACTGAAGAGGTGAAGGAAAAACCTGATTTGGTTGTGGAAGAGA AGGACTCGGATATGAAAGAAGTCAAAAGTGAAGATCAGGCAGATGAGCTG GAACCACCAAAAGAGCCAGTTGAAACACAAGAGAGTGGGACGCCACAAGA T |
|  |  |  |
| ***kat2a*** | **ENSEMBL NO.** | ENSONIG00000001102 |
|  | **NCBI NO.** | XM_025906390.1 |
|  | **Position of CDS in NCBI** | 163-2559 |
|  | **Primer F (5’-3’)** | CACTGACCCTGCTGCTATGC |
|  | **Primer R (5’-3’)** | GTAGGCCAACCAGCCACATC |
|  | **Liver tissue sequences** | AATCTGCTGACACCAAATGCTGCCCGTGATGAGACTGCAAGGCTGGAGGA  GAGACGTGGTATCATAGAGTTTCATGTCATTGGAAACTCACTTTCCCAGA  AATCAAACAAGAAGATCCTGATGTGGCTGGTTGGCCTAC |
|  | **Muscle tissue sequences** | GACTAATCTGCTGACACCAAATGCTGCCCGTGATGAGACTGCAAGGCTGG  AGGAGAGACGTGGTATCATAGAGTTTCATGTCATTGGAAACTCACTTTCC  CAGAAATCAAACAAGAAGATCCTGATGTGGCTGGTTGGCCTAC |
|  |  |  |
| ***kat2b*** | **ENSEMBL NO.** | ENSONIG00000002420 |
|  | **NCBI NO.** | XM_003444058.3 |
|  | **Position of CDS in NCBI** | 493-2871 |
|  | **Primer F (5’-3’)** | GGCCTTTCATGGAGCCTGTG |
|  | **Primer R (5’-3’)** | CTCGCTCTCTGGAGGGTTGT |
|  | **Liver tissue sequences** | CCTGGGTACTACCAAGTCATACGCTTCCCCATGGACCTCAAGACAATGAG TGAGCGTCTAAAGAGCCGGTACTACACGACACGGAAGCTTTTTATGGCCG ACATGCAGCGAATTTTCACAAACTGTCGTGAATACAACCCTCCAGAGAGC GAG |
|  | **Muscle tissue sequences** | ACCTGGGTACTACCAAGTCATACGCTTCCCCATGGACCTCAAGACAATGA GTGAGCGTCTAAAGAGCCGGTACTACACGACACGGAAGCTTTTTATGGCC GACATGCAGCGAATTTTCACAAACTGTCGTGAATACAACCCTCCAGAGAG CGAG |
|  |  |  |
| ***kat6a*** | **ENSEMBL NO.** | ENSONIG00000015837 |
|  | **NCBI NO.** | XM_005472980.3 |
|  | **Position of CDS in NCBI** | 865-7641 |
|  | **Primer F (5’-3’)** | CATCCCGTCCACTGCTTTCC |
|  | **Primer R (5’-3’)** | CCTGTTCACGCTACCACCAC |
|  | **Liver tissue sequences** | TTCTTCCACCTCAGGGCCCCGGGCCCCGCAGTCCCGGCCTCAGGACACGG  ATATGGGTGACAGAGAGGAAGACGATGATGATGAGGAAAGGGAGGAAGAG  GAGTGTGGCAGCATGGGCAGTGGTGGTGGTAGCGTGAACAGG |
|  | **Muscle tissue sequences** | CCTCAGGGCCCCGGGCCCCGCAGTCCCGGCCTCAGGACACGGATATGGGT GACAGAGAGGAAGACGATGATGATGAGGAAAGGGAGGAAGAGGAGTGTGG CAGCATGGGCAGTGGTGGTGGTAGCGTGAACAGG |
|  |  |  |
| ***gtf3c4*** | **ENSEMBL NO.** | ENSONIG00000013016 |
|  | **NCBI NO.** | XM_003440231.5 |
|  | **Position of CDS in NCBI** | 43-2358 |
|  | **Primer F (5’-3’)** | CTTGTGGCGGTTCAAGCTCT |
|  | **Primer R (5’-3’)** | GGCTCGCCTTCCTCTTTCAC |
|  | **Liver tissue sequences** | CTTCACCAGTCACTGCAGTCACCTAACACAGAAAACAACTGGAAACCTGC AAAAGAGGGAAGCAAGGTGTTCATAAGAGATGAGGAAGAGGAGGAGGATG GAGAAGACAAAGAAGATGCTGTGAAAGAGGAAGGCGAGCC |
|  | **Muscle tissue sequences** | CTTCACCAGTCACTGCAGTCACCTAACACAGAAAACAACTGGAAACCTGC AAAAGAGGGAAGCAAGGTGTTCATAAGAGATGAGGAAGAGGAGGAGGATG GAGAAGACAAAGAAGATGCTGTGAAAGAGGAAGGCGAGCC |
|  |  |  |
| ***sirt2*** | **ENSEMBL NO.** | ENSONIG00000016619 |
|  | **NCBI NO.** | XM_003449264.5 |
|  | **Position of CDS in NCBI** | 153-1301 |
|  | **Primer F (5’-3’)** | GCGAGTCTAGTCAGCAGGGT |
|  | **Primer R (5’-3’)** | CCCAGAAGATCAGCTAGAGCCA |
|  | **Liver tissue sequences** | CCCCAGACTGCTCATTAACATGGAGAAAGCAGGGCAGGTTAATCCCATGA TGGGTTTGTTTGGTTTCGGAGAAGGGATGGACTTTGACTCAGACAAGGCT TACAGAGATGTCGCTCACATTAGTACATGTGATGATGGATGTTTGGCTCT AGCTGATCTTCTGGG |
|  | **Muscle tissue sequences** | CCCAGACTGCTCATTAACATGGAGAAAGCAGGGCAGGTTAATCCCATGAT GGGTTTGTTTGGTTTCGGAGAAGGGATGGACTTTGACTCAGACAAGGCTT ACAGAGATGTCGCTCACATTAGTACATGTGATGATGGATGTTTGGCTCTA GCTGATCTTCTGGG |
|  |  |  |
| ***sirt5*** | **ENSEMBL NO.** | ENSONIG00000006380 |
|  | **NCBI NO.** | XM_003457306.5 |
|  | **Position of CDS in NCBI** | 154-1059 |
|  | **Primer F (5’-3’)** | ATTTGCCCAGGTGTGAGCAG |
|  | **Primer R (5’-3’)** | GAGCAAACATGGCTGCAGGA |
|  | **Liver tissue sequences** | TCTCCTTAGACCAGCTGTGGTTTGGTTTGGAGAGACTCTGGATGCTGACA TCCTCACCCGTGCAGAGAAAGTGTTGGACAGCTGTGACCTCTGCCTCGTG GTCGGTACTTCATCCGTCGTGTATCCTGCAGCCATGTTTGCTC |
|  | **Muscle tissue sequences** | GGTCTCCTTAGACCAGCTGTGGTTTGGTTTGGAGAGACTCTGGATGCTGA CATCCTCACCCGTGCAGAGAAAGTGTTGGACAGCTGTGACCTCTGCCTCG TGGTCGGTACTTCATCCGTCGTGTATCCTGCAGCCATGTTTGCTC |
